# Recent Advances in Novel Adsorbents Prepared by the Sol–Gel Method for the Removal of Uranium and Strontium from Radioactive Wastewater: A Review

**DOI:** 10.3390/gels12100891

**Published:** 2026-10-01

**Authors:** Jiayi Jiang, Jiaxue Zhao, Miaoling Chen, Diyun Chen, Minhua Su

**Affiliations:** 1 School of Environmental Science and Engineering, Guangzhou University, Guangzhou 510006, China; jiangiolus@163.com (J.J.);; 2 Center for Water Research, Advanced Institute of Natural Sciences, Beijing Normal University, Zhuhai 519087, China

**Keywords:** uranium, strontium, radioactivity, sol–gel method, adsorption

## Abstract

Uranium- and strontium-containing wastewater from nuclear industries poses a threat to the environment and human health. The sol–gel method, with its tunable composition and synthetic flexibility, is widely used to fabricate novel and high-performance adsorbents. This review covers recent progress in the sol–gel fabrication of UO_2_^2+^ and Sr^2+^ adsorbents, including both inorganic and organic–inorganic hybrid materials. Reported adsorption capacities typically range from about 40 to 670 mg/g for UO_2_^2+^ and from about 40 to 370 mg/g for Sr^2+^, with a few systems achieving even higher values under optimized conditions. Titanates and titanosilicates exhibit outstanding selectivity toward Sr^2+^, whereas phosphate-based materials and amidoxime-functionalized hybrids show strong affinity for UO_2_^2+^. However, practical application is still hampered by high precursor costs, poor shapeability, limited long-term stability, harsh regeneration conditions, and interference from coexisting ions. Future efforts should focus on low-cost materials and shaping processes to promote the engineering applications of tailored materials via the sol–gel method.

## 1. Introduction

Nuclear energy occupies an important position in the global energy structure. Its low carbon emissions and high energy density make it a reliable baseload power source [1]. The development of nuclear energy depends on a sound safety system [2]. However, radioactive wastewater generated by nuclear industries must be properly treated before discharge [3].

Radioactive wastewater comes mainly from reactor operation, fuel reprocessing, and nuclear technology applications. It contains radionuclides such as uranium, strontium, cesium, cobalt, and iodine [4,5]. Among these, uranium and strontium deserve special attention. Uranium has a long half-life and high solubility. It easily accumulates along the food chain and mainly damages the kidneys and bones after entering the human body [6,7]. Strontium behaves chemically like calcium. Once inside the body, it takes part in calcium metabolism and deposits in bone tissue. Long-term irradiation can cause diseases of the blood and bone systems [8,9]. Uranium and strontium are present at low concentrations in wastewater, and their emission limits are very strict. Coexisting ions and complex components make deep removal even harder [10,11].

Radioactive wastewater treatment methods include chemical precipitation, membrane separation, biological treatment, and adsorption, which suffer from limitations including high sludge production, membrane fouling, and microbial inhibition [12]. The adsorption method is easy to operate, removes low-concentration nuclides well, and can be reused. It is widely used in engineering, and its efficiency depends mainly on the structure and surface chemistry of the adsorbent [13,14]. Among many preparation methods, the sol–gel method stands out. It has adjustable composition, controllable structure, and flexible operation. It can mix components uniformly at the molecular scale and can also introduce functional components or perform molding treatment. As a result, it produces adsorption materials with a high specific surface area and abundant active sites [15,16,17]. These sol–gel materials have better component uniformity than those made by coprecipitation. They avoid the high temperature and high pressure needed in the hydrothermal method. They also achieve inorganic-organic molecular-level compositing more easily than the polymerization crosslinking method [18]. Overall, the sol–gel method has clear advantages in controlling structure and morphology, making it suitable for the controllable preparation of functional adsorption materials.

At present, the removal of radionuclides and sol–gel synthesis are mostly studied separately. This review focuses on uranium and strontium, specifically addressing materials prepared by the sol–gel method for their removal. The materials are divided into two types according to the state of the gel network in the final product. The first type is sol–gel materials, in which the gel network formed by hydrolysis-polycondensation is largely preserved, such as xerogels, aerogels, and cryogels. The second type is sol–gel-derived materials, in which the gel network is partly or fully converted by subsequent treatment or by combination with other methods, such as calcination, hydrothermal treatment, and post-grafting, or serves mainly as a shell, interface layer, or binding phase after compositing or coating with other components. Both types originate from a sol–gel process but differ in the extent to which the gel network is retained. The influence of the gel network state on the adsorption behavior will be described when the relevant materials are discussed. The literature was mainly retrieved from the Web of Science Core Collection, supplemented by Google Scholar and the CNKI database. The detailed search strategy is provided in Supplementary Text S1. The retrieved literature was screened according to the topic of this review, i.e., uranium and strontium removal materials prepared by the sol–gel method, and unrelated, similar, or unrepresentative studies were excluded.

On this basis, the materials are divided into inorganic materials and organic–inorganic hybrids. This classification is based on the precursor types and the composition of the final materials, so as to facilitate the later discussion of their adsorption behavior and mechanisms. The former uses metal alkoxides or inorganic salts as precursors. Its adsorption mechanisms mainly involve surface complexation, ion exchange, and redox fixation. The latter is prepared by co-condensation or post-grafting of organosilanes or organic ligands. This introduces functional groups that can selectively coordinate with target ions. The discussion focuses on the mechanisms and applicability of adsorbents. It aims to provide a useful reference for the rational design and optimization of such materials.

## 2. Sol–Gel Method

**Figure 1:**
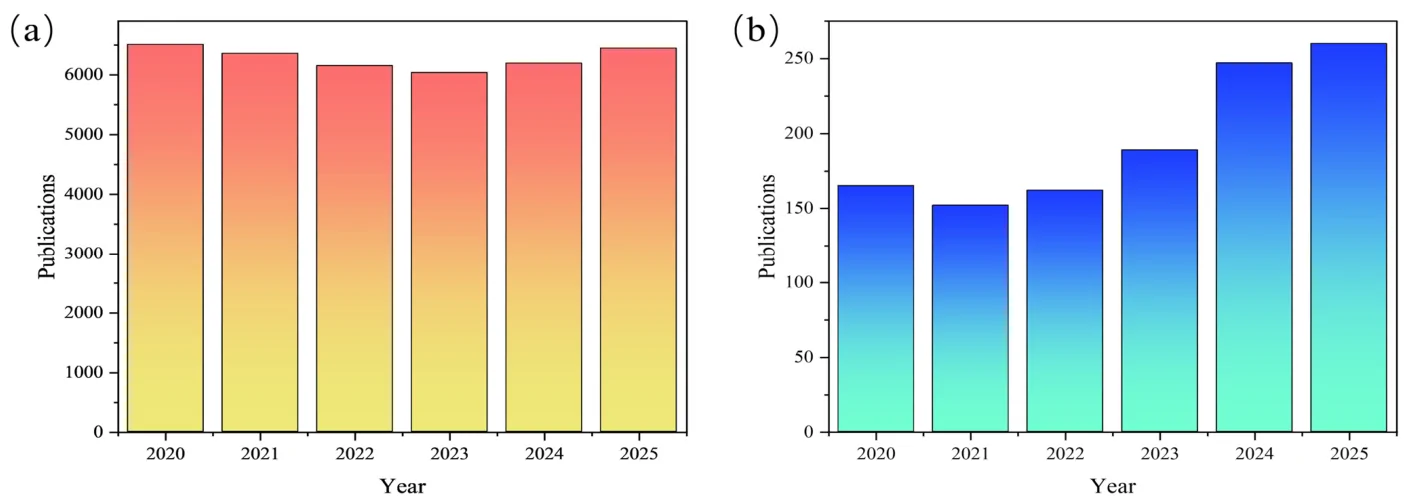
The trend of sol–gel-related papers in Web of Science Core Collection in 2020–2025. (**a**) Total number of publications in the field of sol–gel, search formula: TS = (“sol–gel” OR “sol–gel”). (**b**) Amount of papers published on materials prepared by sol–gel method for radionuclide removal, search formula: TS = ((“sol–gel” OR “sol–gel”) AND (“uranium” OR “strontium” OR “radionuclide” OR “radioactive waste” OR “nuclear waste” OR “radioisotope” OR “cesium” OR “cobalt” OR “radioactive nuclide” OR “radioactive element” OR “actinide”) AND (“adsorption” OR “removal” OR “sorption” OR “separation” OR “uptake” OR “capture” OR “sorbent” OR “adsorb”)).

Based on statistics from the Web of Science Core Collection, the annual number of papers with the theme “sol–gel” remained stable at more than 6000 from 2020 to 2025 (Figure 1a). This indicates that the technology has become highly mature as a material preparation platform. It is worth noting that although the proportion of studies on radionuclide removal is not high, the number of publications has increased significantly, especially in the past three years (2023–2025) (Figure 1b). This reflects growing attention to this preparation technology in radioactive wastewater treatment.

**Figure 2:**
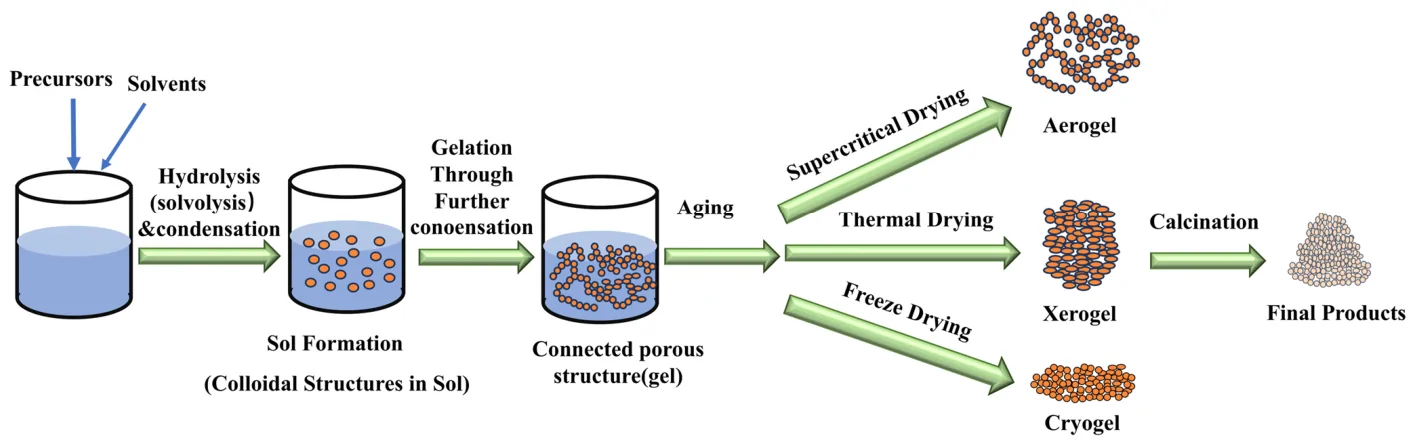
Schematic of the sol–gel method for the fabrication of materials.

Generally, the sol–gel method [15,16,17] uses metal alkoxides or inorganic salts as precursors to form a gel network through liquid-phase hydrolysis-condensation, followed by aging, drying, and heat treatment to achieve controllable preparation of polymorphic products ranging from powder to bulk (Figure 2). The liquid-phase reaction provides molecular-level homogeneity of the components, while the product structure can be controlled to a certain extent by adjusting the pH, temperature, or precursor ratio [19]. This route has mild conditions and wide precursor compatibility. It also allows functional components or organic groups to be introduced in situ while keeping their activity. The wet gel drying path determines the final state: supercritical drying gives aerogels, atmospheric-pressure drying gives xerogels, and freeze-drying gives cryogels [20,21]. A pure inorganic gel tends to shrink and crack during drying. Introducing organic components can toughen the skeleton and inhibit shrinkage [22,23]. A xerogel can be densified or crystallized by heat treatment. A sol can also be coated on a substrate to form a film [24].

Process variables such as gelation time, aging, drying, and heat treatment, together with the post-treatment scheme, determine the final structure of the adsorbent, and these variables are interdependent [15,16,17]. The gelation time depends on the pH, temperature, precursor concentration, water-to-precursor ratio, type and amount of catalyst, solvent, and nature of the precursor; additives such as chelating agents can also lengthen it by slowing down hydrolysis [21]. Prolonged aging consolidates the gel network through continued condensation, improving the uniformity of the pore structure and increasing the mechanical strength of the network [17]. The effect of aging on the BET surface area, however, depends on the specific system [25]. Supercritical drying preserves the pore structure to a greater extent, whereas atmospheric-pressure drying and freeze-drying tend to cause pore collapse or to produce mechanically weaker networks [15,16]. Heat treatment can improve crystallinity and stabilize the framework, but excessive calcination may sinter the pores and remove surface functional groups, reducing the number of accessible adsorption sites [16]. Taken together, these process variables must be balanced to optimize site accessibility, pore structure, and structural stability for a given application. At the macroscopic level, the same variables and the post-treatment scheme also determine the product morphology, which can be adapted to different engineering requirements such as adsorption, fixed-bed operation, and electrocatalysis [26,27].

The basic reaction paths of hydrolysis and polycondensation are as follows:

### 2.1. Hydrolysis Reaction

Taking M(OR)_n_ (M is a metal, R is an alkyl group) as an example, it is hydrolyzed under the action of water and a catalyst.

M(OR)_n_ + nH_2_O→M(OH)_n_ + nROH (1)

Equation (1) represents an idealized complete hydrolysis, in which all OR groups are replaced by OH groups. In practice, hydrolysis is often partial, and condensation reactions (2) and (3) can begin before hydrolysis is complete. As a result, partially hydrolyzed species such as M(OR)_n−x_(OH)_x_ are common intermediates, and the final gel network typically contains both unhydrolyzed OR groups and condensed M–O–M bonds.

### 2.2. Condensation Reaction

Condensation through dehydration or alcohol loss to form a M–O–M bridge bond network:

Lost water polycondensation: M–OH + HO–M→M–O–M + H_2_O (2)

Lost alcohol polycondensation: M–OR + HO–M→M–O–M + ROH(3)

The traditional sol–gel method mostly relies on metal alkoxide precursors, and some systems are easy to precipitate rather than gel, which is not conducive to large-scale production. Strelko et al. [28] used cheap inorganic salts instead of alkoxides and introduced competitive ions to inhibit the rapid formation of the solid phase, making the system gel. The competitive ions can be removed by acid washing after gelation, leaving controllable pores. This strategy can be combined with the oil drop method or the gas drop method to prepare millimeter-scale spherical particle adsorbents, which can overcome the problems of easy loss of powder materials and large pressure drop [29].

## 3. Characteristics and Hazards of U and Sr Radionuclides

Before discussing the characteristics and hazards of uranium and strontium, the terminology used in this review should be clarified. “U” and “Sr” refer to the elements. In an aqueous solution, uranium predominantly exists as the uranyl ion, UO_2_^2+^, and strontium as the free ion, Sr^2+^. Most adsorption studies use UO_2_^2+^ and Sr^2+^ as chemical analogs of the corresponding radioactive isotopes, such as ^238^U/^235^U and ^90^Sr. When radioactive isotopes are specifically discussed, the isotope notation is used.

### 3.1. Uranium

Uranium is an actinide element. It mainly has three radioactive isotopes in nature, viz., ^234^U, ^235^U, and ^238^U [30]. The speciation of UO_2_^2+^ in an aqueous solution depends strongly on solution conditions such as pH, total uranium concentration, dissolved inorganic carbon, and other factors. Because UO_2_^2+^ is a strong Lewis acid, it can complex with many different compounds via oxygen atoms, especially in natural waters. Under oxidizing conditions, UO_2_^2+^ is predominant at low pH (pH < 5) [31]. At dilute concentrations (<10^−6^ M), the dominant hydrolyzed species is the mononuclear UO_2_(OH)^+^, which forms before polynuclear complexes and UO_2_(OH)_2_ become significant. Above this concentration, a mixture of UO_2_(OH)_2_, UO_2_(OH)_4_^2−^, and UO_2_(OH)_5_^3−^ is also observed [32,33]. In the (CH_3_)_4_N–OH media, at a total uranium concentration of 0.05 M, two polynuclear hydroxo complexes, (UO_2_)_2_(OH)_2_^2+^ and (UO_2_)_3_(OH)_5_^+^, dominate at pH 3–4, whereas schoepite (UO_2_(OH)_2_·H_2_O) precipitates at pH 6–11 [34]. In seawater, uranium exists mainly as carbonate complexes such as UO_2_(CO_3_)_3_^4−^ and UO_2_(CO_3_)_2_^2−^ [32,33]. Under reducing conditions, UO_2_^2+^ is reduced and forms insoluble UO_2_ precipitates [32,33].

Uranium pollution occurs throughout the nuclear fuel cycle. Uranium-containing wastewater is generated by front-end mining and hydrometallurgy, trace uranium is released by inner cladding damage, and long-term contamination results from nuclear tests and submarine operations [35,36]. The development of associated minerals, such as rare earth smelting and phosphate rock processing, also enriches uranium in waste residues and wastewater [37].

Uranium has both radioactive and chemical toxicity. Its decay daughters release α, β, and γ rays, and the high ionization density of α particles causes intensive damage to cells [38]. Accumulation of UO_2_^2+^ in renal tubules can cause renal tubular injury and renal failure. Long-term low-dose exposure is also associated with bone toxicity, reproductive toxicity, genotoxicity, and increased risk of malignant tumors [39,40].

### 3.2. Strontium

The fission of ^235^U and ^239^Pu produces a variety of radioactive products [41], and ^90^Sr has attracted much attention due to its high yield (approximately 5.8% for ^235^U and 2.1% for ^239^Pu by thermal neutrons) and long half-life (28.8 years) [42]. Its β-rays can be continuously released for hundreds of years, posing a medium- and long-term environmental risk [43].

Sr is often present as hydrated Sr^2+^ in water. However, its complexation with CO_3_^2−^ and SO_4_^2−^, as well as the precipitation of SrCO_3_, depends on solution composition and saturation state [44,45]. In the presence of CO_3_^2−^, soluble complexes such as SrCO_3_(aq) can form. SrCO_3_ precipitation may occur when the solubility product is exceeded, especially in alkaline solutions with a high carbonate concentration. At high pH, hydrolyzed species such as [SrOH]^+^ and [Sr(OH)_2_]^0^ become increasingly important [44,46]. These changes in speciation affect the migration behavior and biological accessibility of Sr^2+^. They also influence its adsorption behavior because different Sr species may interact differently with adsorbent surfaces [45,46].

Sr^2+^ and Ca^2+^ belong to the alkaline earth metals with similar ionic radius and charge, and their chemical behaviors are highly similar [8,9]. Organisms tend to mistake it for Ca^2+^ and deposit it in the bones through the calcium metabolic pathway, which is enriched along the food chain. The continuous release of β-rays in the bones significantly increases the risk of leukemia and osteosarcoma [47,48]. Animal experiments have shown that it can pass through the placental barrier to cause developmental malformations [49].

^90^Sr has a wide range of sources, including nuclear reactor operation, nuclear weapon tests, and nuclear accidents and activities [50]. From 1945 to 1980, a total of 622 PBq of ^90^Sr was released during the global nuclear tests. Chernobyl, Fukushima and other accidents also released a large amount of ^90^Sr, which is still retained in the environment for a long time [43,51]. ^90^Sr in nuclear wastewater can be diffused into the ocean with water flow and enriched through the food chain. If the ^90^Sr radioactive source used in industrial flaw detection and medical radiotherapy is not handled properly, it becomes a local pollution source [52].

## 4. Materials for U and Sr Removal

Most studies reviewed in this section use model solutions. These solutions often contain single ions or simple simulated matrices and are not real radioactive wastewater. Model-solution results reflect intrinsic adsorption behavior; however, they may differ from results obtained in real radioactive wastewater.

### 4.1. Inorganic and Composite Materials

#### 4.1.1. Iron Oxides

Iron oxides mainly include magnetite (Fe_3_O_4_) and maghemite (γ-Fe_2_O_3_). They exhibit superparamagnetism and can be easily synthesized; therefore, they are the most commonly used materials for preparing magnetic adsorbents by the sol–gel method [53]. Using ferric alkoxides or inorganic salts as precursors, the particle size and magnetic responsiveness can be controlled by hydrolysis-condensation [5,7]. Nanoscale iron oxides are rapidly magnetized and aggregated under an external magnetic field. After removing the magnetic field, they can be redispersed without remanence. Thus, solid-liquid separation can be achieved without centrifugation or filtration. However, bare iron oxide tends to agglomerate and possesses limited active sites, which restricts selective adsorption [5,6,7]. To address this, researchers have extended the sol–gel method to core-shell structures. The magnetic core can be obtained by coprecipitation or hydrothermal methods, followed by coating with a functional shell via the sol–gel method. This core-shell design separates magnetic separation from adsorption.

Papynov et al. [54] used FeCl_3_·6H_2_O as the iron source and butyl silicone latex as a pore-forming template to prepare porous γ-Fe_2_O_3_/α-Fe_2_O_3_ precursors. These were then reduced in a CO gas flow at 900 °C to obtain Fe^0^/γ-Fe_2_O_3_ composites. After reduction, the specific surface area dropped from 47.3 m^2^/g to 0.2 m^2^/g; however, introducing nano zero-valent iron boosted the saturation magnetization to 140 emu/g. In acidic and mildly acidic media, the removal rate of UO_2_^2+^ was nearly 100%. This combination of crystal phases enables a redox adsorption mechanism, forming insoluble UO_2_ on the sorbent surface, as confirmed by XPS (prevalence of U^4+^) and EDX (local uranium content reaching 80 wt.%). The sharp drop in surface area was accompanied by the strong reduction and fixation ability of the Fe^0^ active phase, while the magnetic recovery feature was retained.

Perovskite-type composite oxides with Fe as the functional core can be obtained by adjusting the precursor composition and heat treatment conditions. Li et al. [55] used citric acid and ethylenediaminetetraacetic acid as complexing agents to obtain perovskite-type Ba_0_._5_Sr_0_._5_FeO_3−_δ by sol–gel calcination. When the adsorbent dosage was 0.8 g/L, the removal rate of uranium from 10 mg/L uranium-containing wastewater reached 97.38%, and adsorption equilibrium was reached within 15 min. XPS analysis showed that part of UO_2_^2+^ was reduced to U^4+^ by Fe^2+^, with U^4+^ and UO_2_^2+^ accounting for 65.53% and 34.47% of the adsorbed uranium, respectively. Meanwhile, Fe^2+^ was oxidized to Fe^3+^/Fe^4+^. After four adsorption–desorption cycles, the removal efficiency was still 86.15%, and the desorption efficiency was 83.03%, indicating good reusability.

**Figure 3:**
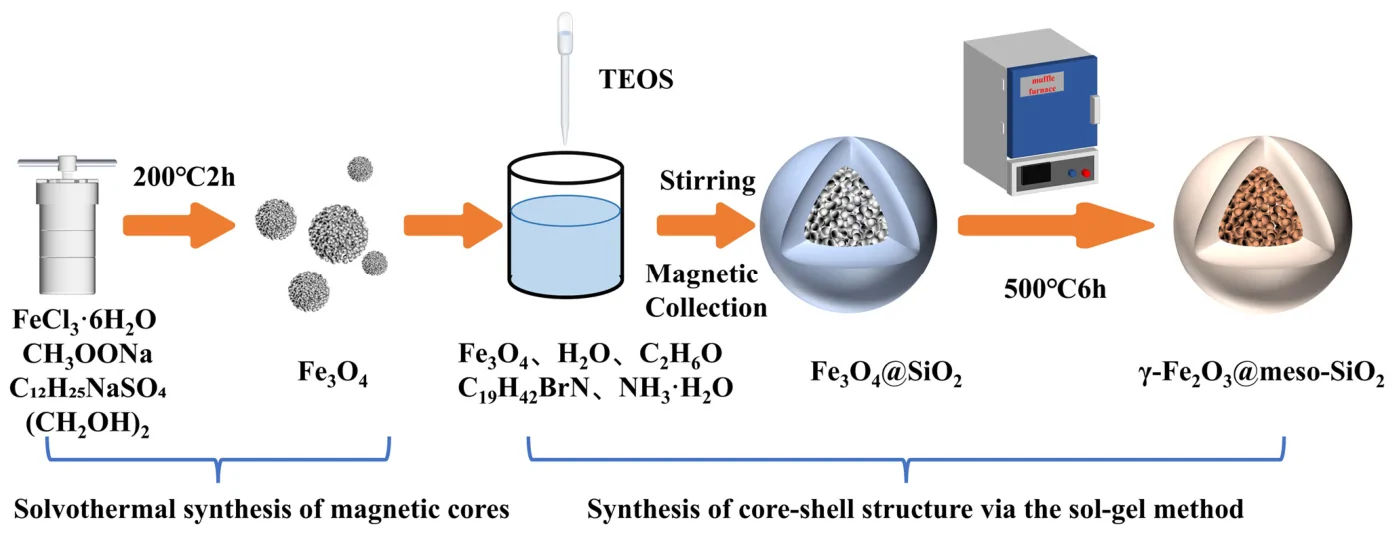
Schematic of core-shell structure prepared by sol–gel method [56].

The value of the core-shell structure lies in the tunability of the shell function. The γ-Fe_2_O_3_@meso-SiO_2_ core-shell microspheres (Figure 3) prepared by Li et al. [56] showed that UO_2_^2+^ can be captured through complexation and electrostatic attraction with mesoporous SiO_2_ channels and surface Si–OH under weakly acidic conditions. Adsorption equilibrium was reached within 15 min, and the efficiency remained above 90% after five cycles. The adsorption efficiency of UO_2_^2+^ was still more than 90% in complex water containing K^+^, Na^+^, Mg^2+^, Pb^2+^, and other competitive ions. The adsorption efficiency of Sr^2+^ and Cs^+^ was extremely low, indicating good selectivity. Yi et al. [57] synthesized Fe_3_O_4_@SiO_2_@TiO_2_ and then converted TiO_2_ into Fe_3_O_4_@titanate fiber by hydrothermal treatment with concentrated NaOH. The SiO_2_ interlayer plays a key role in anchoring titanate fibers on the surface of the magnetic cores. By adjusting the synthesis conditions, the shell morphology can be switched between fibrous and flake forms, confirming the high adjustability of the shell structure and function. The material has an adsorption capacity of 37.1 mg/g for Sr^2+^ and reaches equilibrium in 30 min. It also shows better selectivity in simulated wastewater. The desorption rate was 92.2% with 5% ethylenediaminetetraacetic acid disodium salt.

In the studies reviewed in this subsection, iron oxides serve both as adsorbents and, more commonly, as the magnetic core or functional phase of sol–gel-derived composites. Single iron oxides tend to agglomerate and offer limited active sites, so most work uses them in the core-shell or composite form. The iron source and the subsequent reduction or coating steps determine which active phase or surface group dominates the material, and these groups correspond to different uptake behavior [54,56]. The magnetic component mainly provides recoverability, while the active phase governs the uptake. Thus, the reported capacities depend more on the shell chemistry and composite architecture than on the iron oxide itself. The sharp drop in BET area after reduction, together with the retained UO_2_^2+^ removal, suggests that surface area alone is not the controlling factor in these systems [54].

#### 4.1.2. Titanium Oxides

Titanium oxide (mainly TiO_2_) has a basic structural unit of a TiO_2_ octahedron, good chemical stability and photocatalytic activity, and is not easily degraded in acidic waste liquid or under light irradiation [58]. In the sol–gel method, tetrabutyl titanate, titanium tetraisopropoxide, and other precursors can be used as titanium sources, and different crystal phases can be obtained by adjusting the calcination temperature [59,60].

The surface Ti–OH sites provide stable complexation anchor points for UO_2_^2+^. Odeh et al. [61] prepared TiO_2_ nanoparticles by a sol–gel-hydrothermal method. With an initial uranium concentration of 100 ppm, a solution volume of 20 mL, an adsorbent dose of 10 mg, a contact time of 4 h, and a constant temperature, the final concentration at pH 9.0 was 7.76 ppm, giving a UO_2_^2+^ removal rate of 92.24%. Its performance is closely related to the anatase crystal phase and to the density of Ti–OH sites on the surface. However, TiO_2_ particles tend to agglomerate, resulting in a decrease in active-site density and limited adsorption capacity [62].

There is no simple positive correlation between specific surface area and adsorption capacity. Kimling et al. [63] studied crystalline and amorphous TiO_2_/ZrO_2_ porous microspheres. The crystalline microspheres had a lower specific surface area (52–59 m^2^/g) than the amorphous ones (95–247 m^2^/g), yet showed a higher adsorption capacity for UO_2_^2+^ (0.170 mmol/g, about 40.25 mg/g). This value was derived from the Langmuir isotherm model (R^2^ = 0.92) at pH 3.8 with an initial UO_2_^2+^ concentration of 10–500 ppm. The isotherm showed a steep initial rise followed by a plateau at C_0_ = 200–300 ppm, where uptake reached about 99%, consistent with monolayer coverage. The pseudo-second-order kinetic model also fitted the data well (R^2^ = 0.988). The higher capacity was attributed to the higher surface hydroxyl density and larger mesopore size of the crystalline microspheres, indicating that the density of accessible surface Ti–OH is a key factor in uranium adsorption.

Building on the aforementioned titanium-based binary oxide research, Ding et al. [64] prepared titanium–aluminum bimetallic oxides loaded on graphene oxide (GO@Ti_x_Al_1−x_Oᵧ). The introduction of GO improved the dispersion of the bimetallic oxide nanoparticles, allowing the oxygen-containing functional groups on GO to form multiple coordination interactions with Ti/Al–OH sites. In static adsorption experiments, the Langmuir isotherm model (R^2^ > 0.99) gave a maximum UO_2_^2+^ adsorption capacity of 614.6 mg/g for this composite at pH 4, 298 K, and an initial concentration of 5–80 mg/L. Adsorption equilibrium was reached within 60 min. In dynamic adsorption experiments, the Thomas model gave a capacity of 610.3 mg/g, close to the Langmuir value, with a breakthrough time of 600 min. After five adsorption column cycles, the breakthrough time remained at 480 min, and the removal efficiency remained above 90%.

**Figure 4:**
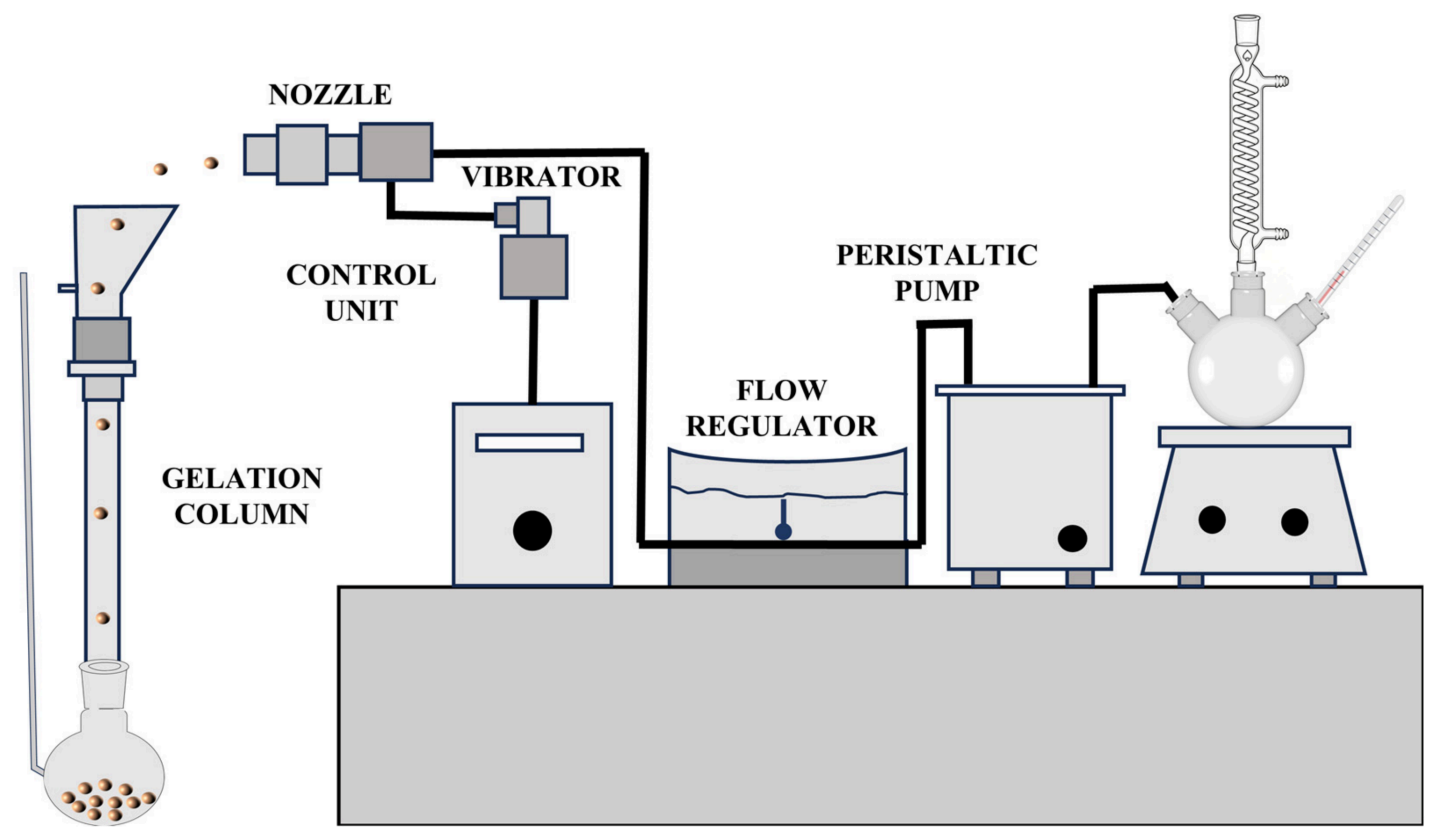
Schematic diagram of gel-forming column device [66].

For Sr adsorption, Mironyuk et al. [65] prepared carbonate-modified mesoporous carbonated TiO_2_ nanoparticles using Na_2_CO_3_ modification. The carbonate chemically adsorbs on the TiO_2_ surface in a bidentate coordination form (=Ti(O_2_CO)), giving a specific surface area of 379.9 m^2^/g. The experimental equilibrium adsorption capacity for Sr^2+^ reaches 204.4 mg/g, much higher than that of unmodified TiO_2_ (70.9 mg/g). The sol–gel method can also be combined with shaping-oriented methods. Tel et al. [66] used ZrOCl_2_ and TiCl_4_ as precursors and ammonia water as a gelling agent. They prepared ZrO_2_ and ZrO_2_–TiO_2_ composite oxide microspheres via a gel-forming method (Figure 4). The Sr^2+^ sorption data were fitted with the Freundlich, Langmuir, and Dubinin-Radushkevich isotherm models. However, these models only describe the equilibrium distribution of Sr^2+^ under their respective assumptions. The Langmuir capacity of 28.01 mg/g is therefore a model-fitted value, not a directly measured one. In addition, the experimental isotherm tends to level off at higher concentrations, but the available data are insufficient to confirm a plateau. The Dubinin-Radushkevich mean sorption energies were 8.84 kJ/mol for ZrO_2_ and 8.01 kJ/mol for ZrO_2_–TiO_2_. The authors interpreted these values as indicating ion exchange. However, these values fall within the borderline range between ion exchange and physical adsorption and therefore do not conclusively confirm an ion-exchange mechanism.

In addition, the sol–gel method can couple the photocatalytic properties of TiO_2_ with adsorption. Wang et al. [67] combined it with the impregnation method to prepare a WO_3_-doped TiO_2_ foam nickel electrode (TiO_2_/WO_3_@NF). The introduction of WO_3_ forms a Z-type heterojunction, achieving a 97.75% removal rate of 10 mg/L UO_2_^2+^ within 3 h, which is much better than the pure TiO_2_ electrode (83.3%). Mechanistic studies show that photogenerated electrons (e^−^) and superoxide radicals (•O_2_^−^) are the main active species responsible for reducing UO_2_^2+^, which is fixed on the electrode surface as UO_2_ and UO_2_O_2_H_2_O. Performance depended strongly on preparation and operating conditions. WO_3_ doping was varied from 5 to 25 wt%, and the removal efficiency first increased and then decreased, peaking at 10 wt%. This is because excess WO_3_ reduced its dispersion and blocked the porous nickel foam. The heat treatment temperature was also optimized, and 80 °C gave the best removal among 50, 80, 100, and 200 °C. Removal increased with applied voltage, reaching 97.75% at 2 V. The highest removal was observed at pH 3 (97%), and it dropped at pH 2 (83%) and at pH 4–7, which was attributed to hydrolyzed uranyl complexes. The incident light wavelength also mattered: light at 420 and 500 nm gave better reduction than light at 400 nm. These results show that such immobilized TiO_2_-based photoelectrodes are governed by both preparation and operating conditions. Table 1 summarizes the performance comparison of titanium oxide materials in adsorbing uranium and strontium.

Different synthesis conditions can yield crystalline or amorphous materials, and the pore structures formed in these materials further influence the adsorption performance [63]. Synthesis conditions and post-treatment can affect the surface hydroxyl population, and the density of accessible Ti–OH sites, rather than the total surface area, appears to be most closely associated with the adsorption performance [63]. Modification and compositing can further adjust this surface-site density [64], but their effect has so far been assessed mainly through capacity changes rather than by the direct measurement of accessible sites [65]. For TiO_2_-based photoelectrodes, the material does not rely on surface complexation alone but uses photogenerated charge carriers to reduce UO_2_^2+^, and its performance depends on both the preparation conditions and the operating parameters [67].

#### 4.1.3. Titanates

Titanates are formed by TiO_6_ octahedra connected through shared edges or vertices, and the framework charge is balanced by alkali metal ions (e.g., Na^+^ and K^+^) in the interlayers or channels, giving them reversible ion-exchange ability [68,69]. Unlike the surface hydroxyl complexation mechanism of titanium oxides, titanates show unique size-sieving and ion-exchange selectivity toward alkaline earth metal ions, especially Sr^2+^ [70].

The ion-exchange ability of layered titanates comes from the exchangeable Na^+^ between layers. Sodium titanate is usually prepared by the sol–gel method, using tetrabutyl titanate and sodium nitrate as precursors, with a complexing agent added to slow down alkoxide hydrolysis [71]. A uniform sol is obtained by adjusting the water-to-titanium ratio and aging conditions. After drying, the layered structure is formed by calcination at a suitable temperature, and temperature control is key to obtaining a regular layered phase [71]. Li et al. [71] found that the precursor remains amorphous at 300 °C. At 400 °C, it starts to form the layered sodium titanate (Na_2_Ti_n_O_2n+1_) crystal phase. At 500–600 °C, rutile grows. Above 800 °C, it easily transforms into a three-dimensional perovskite phase, which causes the layers to collapse and leads to a loss of ion-exchange capability. Layered sodium titanate calcined at 400 °C has a maximum adsorption capacity of 49.6 mg/g for Sr^2+^ and shows high selectivity in the presence of coexisting ions such as Ba^2+^, Ca^2+^, Mg^2+^, Cs^+^, and K^+^. After loading Sr^2+^, calcination at 1100 °C can convert it into a leach-resistant titanate ceramic, turning the adsorbed ions into a stabilized, solid form [71].

Kim et al. [72] used an improved medium-temperature sol–gel method to synthesize amorphous sodium titanate. In a system where Sr^2+^ and Cs^+^ coexist, it shows very high ion-exchange selectivity: within 1 h, the Sr^2+^ removal rate reaches 99.9%, with a maximum adsorption capacity of 193.93 mg/g, while the Cs^+^ removal rate is only 40.67%, giving a Sr/Cs selectivity of over 150. This selectivity can be attributed to two factors. First, the ionic radius of Sr^2+^ (125 pm) is considerably smaller than that of Cs^+^ (173 pm), allowing Sr^2+^ to access adsorption sites that are less accessible to the larger Cs^+^. Second, the divalent charge of Sr^2+^ provides stronger electrostatic attraction to the negatively charged titanate framework than monovalent Cs^+^. XPS analysis confirmed that Sr^2+^ was adsorbed via ion exchange with Na^+^, followed by surface precipitation to form a SrTiO_3_-like structure.

**Figure 5:**
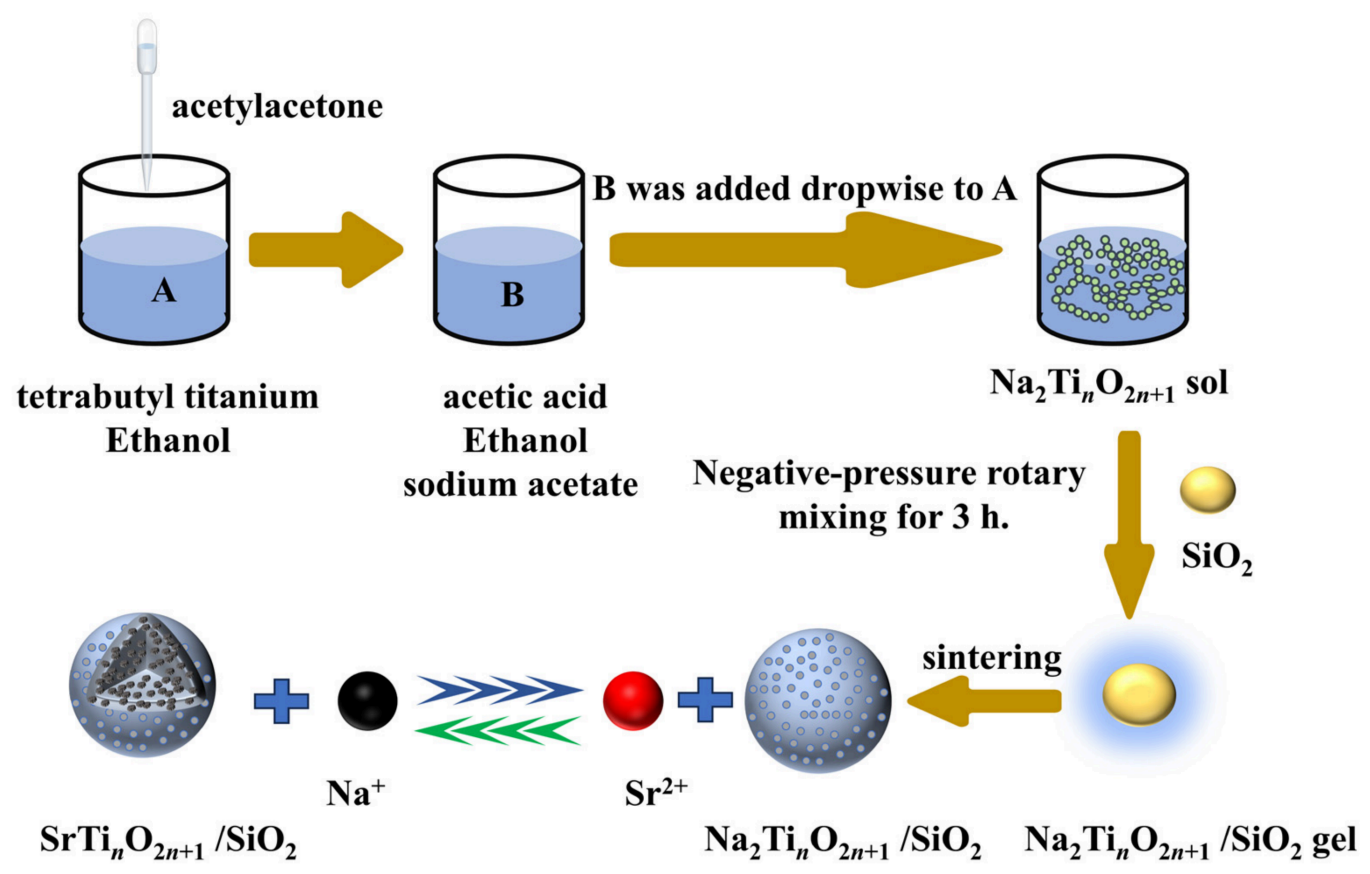
Preparation of Na_2_Ti_n_O_2n+1_/SiO_2_ and schematic diagram of Sr^2+^ adsorption [73].

To make shaping and column use easier, Liu et al. [73] loaded sodium titanate into SiO_2_ channels and calcined it to obtain a silica-based sodium titanate composite adsorbent (Na_2_Ti_n_O_2n+1_/SiO_2_) (Figure 5). The material has a regular spherical morphology with a diameter of 75–100 μm, a BET surface area of 44.83 m^2^/g, and a mesoporous structure with a mean pore diameter of 62.33 nm. The static Sr^2+^ adsorption capacity was 33.31 mg/g at 25 °C, and equilibrium was reached within 2 min. In a fixed-bed column (5 mm i.d., 100 mm bed height, 0.95 g adsorbent, 2 mL bed volume, 1 mL/min, 30 BV/h), 950 bed volumes of simulated seawater were treated over about 165 h without detectable strontium breakthrough. These results indicate that the material is promising for column operation, but its long-term stability and scale-up behavior have not yet been verified.

Titanate with a three-dimensional tunnel structure can also be prepared by the sol–gel method. Linggen et al. [74] synthesized a hierarchical mesoporous cerium titanate (CeTi_2_O_6_) with a brannerite structure by combining soft and hard dual templates (glucose as the soft template and colloidal SiO_2_ as the hard template). The material had a high specific surface area (up to ~100 m^2^/g) and a large pore volume (up to ~0.4 cm^3^/g), with 5–50 nm mesopores accounting for 84–98% of the total pore volume. The Langmuir-fitted monolayer UO_2_^2+^ adsorption capacity reached about 53 mg/g. This fitted value is supported by several lines of evidence. It shows a linear relationship with the specific surface area of the powders and also with the surface hydroxyl concentration. Within 15 min, UO_2_^2+^ adsorption reaches over 70% of the equilibrium capacity, indicating good accessibility of adsorption sites. The material also remains stable in ~2 mol/L acidic and basic solutions.

The selectivity of titanates is governed by the exchangeable cations and the geometry of the interlayer or channel space [68,69,70]. The hydrated ionic radius determines the effective size of the ion in solution. It is the hydrated species, not the bare ion, that enters the interlayer galleries or framework channels [71,74]. A smaller hydrated radius is geometrically more favorable for entering narrow spaces [71,74]. The accessible spacing is set by the synthesis temperature for layered titanates and by the framework topology for tunnel-structured titanates. It defines the height or diameter available for ion entry and therefore the maximum size of the hydrated ion that can be accommodated [67,70]. Size sieving arises from the match between the hydrated radius and the accessible spacing. Cations whose hydrated size fits the available space exchange readily with the exchangeable cations, whereas larger hydrated species are excluded or exchange more slowly [71,72,74].

Overall, the selectivity of titanates is governed by both geometric and charge effects. Layered titanates rely on interlayer spacing and size matching, but their selectivity can be lost at high calcination temperatures because of interlayer collapse and phase transformation [72]. Amorphous sodium titanate lacks well-defined channels, so its selectivity mainly arises from ionic radius and charge [72]. Tunnel-structured titanates offer more stable framework channels and can also accommodate UO_2_^2+^ through size matching [74]. In practical use, Ca^2+^ and Mg^2+^ compete with Sr^2+^ for the same exchange sites, and Ca^2+^ interferes more strongly because its ionic radius is closer to that of Sr^2+^ [72]. Shaping into pellets or SiO_2_-supported spheres improves column compatibility but may reduce the intrinsic Sr^2+^ capacity relative to the powder form. Long-term stability and scale-up behavior remain to be verified [73].

#### 4.1.4. Titanosilicates

Unlike the pure titanate framework, titanosilicate is characterized by a mixed framework composed of TiO_6_ octahedra and SiO_4_ tetrahedra, and Si is the structural component of the framework rather than the external carrier. This mixed framework has both the ion-exchange capacity of titanate and the structural stability of silicate [75,76].

Titanosilicates are usually prepared by the sol–gel method, using titanium salts and silicon salts as co-precursors. The Ti–O–Si network forms directly through hydrolysis and polycondensation. Oleksiienko et al. [77] synthesized amorphous titanosilicate (TiSi). TiSi(p), made from pure precursors, has micro- and mesoporous structures with a specific surface area of 270.3 m^2^/g. Industrial-grade TiSi(t) has a hierarchical structure with micro-, meso-, and macropores and a surface area of 158.7 m^2^/g. The maximum adsorption capacity for Sr^2+^ is 1.95 mmol/g for TiSi(p) and 1.42 mmol/g for TiSi(t). Both maintain high removal efficiency in the pH range of 4–12 and reach equilibrium in 30–60 min. Adsorption is driven mainly by surface ion-exchange sites (≡Ti–O–Si–ONa), followed by the formation of SrCO_3_ heterogeneous crystals on the surface. This material can be used directly without granulation, showing the shaping advantages of the sol–gel method.

By combining hydrothermal post-treatment, crystalline titanium silicate can be further obtained. Pian et al. [78] prepared sodium titanosilicate (Na_2_TiOSiO_4_) using tetrabutyl titanate and sodium silicate as precursors. By adjusting the alkali-to-silicon ratio and hydrothermal time, they obtained three morphologies: almond-shaped, small almond-shaped (Almond-b), and walnut-shaped. The walnut-shaped sample had the highest specific surface area (7.126 m^2^/g) and the most negative zeta potential (−37.4 mV), giving it the best adsorption performance among the three. In an aqueous solution, their adsorption capacities reached 220, 195, and 237 mg/g within 1 min. At low concentrations, adsorption occurs mainly through electrostatic interactions, along with exchange between interlayer Na^+^ and UO_2_^2+^. At higher concentrations, pre-adsorbed UO_2_^2+^ acts as nucleation centers, prompting uranium crystals to form on the surface, which keeps the material highly efficient for uranium removal even in concentrated solutions. In simulated seawater (pH = 8.3), the saturation capacities reached 208, 256, and 266 mg/g. The removal rate exceeded 60% at a seawater uranium concentration of 3.3 μg/L and over 90% when the uranium concentration was above 50 μg/L. The material can be regenerated in 0.1 mol/L HNO_3_ and shows good selectivity for UO_2_^2+^ in multi-ion competitive systems.

Crystalline titanosilicates with a three-dimensional channel structure can also be obtained through a sol–gel-hydrothermal route. Wang et al. [79] used Na_2_Si_2_O_3_·9H_2_O and TiCl_4_ as the silicon and titanium sources to synthesize nanocrystalline silicotitanate (nano-CST) via the sol–gel-hydrothermal method. Under certain conditions, pure CST crystals with an average particle size of about 46.3 nm can be obtained. The adsorption equilibrium time for Sr^2+^ is only 4 h, with distribution coefficients reaching up to 1.9 × 10^6^ mL/g. SEM-EDS confirmed that Sr^2+^ exchanged with interlayer Na^+^. The material remains stable across a wide pH range, and the fully inorganic synthesis requires no templates.

The titanosilicate work reviewed here shows that the precursor purity and hydrothermal treatment control the pore hierarchy and the surface charge [77,78,79]. These two factors together determine how many exchange sites remain accessible. The pore hierarchy obtained from different precursors influences the Sr^2+^ capacity, and the pure-precursor sample outperforms the industrial-grade one in the reported comparison [77]. Crystalline titanosilicates obtained by hydrothermal post-treatment show higher UO_2_^2+^ capacities. Their morphology also matters, which suggests that surface area and surface potential, not framework composition alone, are involved. The uptake mechanism changes with concentration. At low concentration, electrostatic interaction and interlayer exchange dominate. At high concentration, surface crystallization becomes important [78].

#### 4.1.5. Metal Phosphates

Metal phosphate (MP) materials have open layered or 3D framework structures, formed when metal ions link with phosphate groups (PO_4_^3−^) through corner-sharing or edge-sharing. According to hard and soft acids and bases theory, Sr^2+^, as an alkaline earth metal ion, can form stable ionic bonds with PO_4_^3−^; UO_2_^2+^ can form coordination bonds with PO_4_^3−^ to remain stable [80,81].

Meng et al. [82] combined the sol–gel method with supercritical drying to prepare three-dimensional mesoporous titanium phosphate (TiP) aerogels. The resulting aerogel has a specific surface area of 136.33 m^2^/g and a phosphate-rich surface. Its three-dimensional porous network shortens the mass transfer path, while the phosphate groups show a strong affinity for Sr^2+^. At an initial concentration of 300 mg/L, 25 °C, and pH 5, the adsorption capacity reached 216.2 mg/g. The isotherm data were well fitted by the Langmuir model (R^2^ = 0.991), which gave a theoretical maximum capacity of 373.6 mg/g, consistent with the D-R model (365.0 mg/g). Equilibrium was reached within 10 min. The distribution coefficient was 4740.03 mL/g, and the removal rate exceeded 80% in seawater, lake water, and tap water. XPS and Raman confirmed that Sr^2+^ forms strong ionic bonds with phosphate groups. The mechanism involves a synergy between electrostatic interaction and ion exchange.

Similarly, Chen et al. [83] prepared porous zirconium phosphate (ZrP) aerogels by post-modification and phosphorylation of ZrO_2_ aerogels in Na_2_HPO_4_ solution. The material retains the mesoporous structure of the ZrO_2_ aerogel, and the specific surface area is 98.14 m^2^/g. The phosphate groups are evenly distributed in the pores, and the interlayer Na^+^ provides abundant ion-exchange sites. The static adsorption capacity of Sr^2+^ was 198.44 mg/g, about 90% of Sr^2+^ was removed within 10 min, and the equilibrium time was 50 min. The removal rate of low-concentration Sr^2+^ in seawater by the dynamic column experiment was more than 99%, and the Thomas model fitting capacity (185.95 mg/g) was in good agreement with the static value. To verify practical use, they added ^88^Sr to seawater to simulate nuclear wastewater and pumped it into a packed column; before the effluent volume reached 1740 mL, the removal efficiency of ^88^Sr exceeded 99%. Since isotopes have the same chemical behavior, if ^88^Sr serves as a carrier for ^90^Sr or other radioactive strontium, the removal efficiency of radioactive strontium should also exceed 99%. The breakthrough curve for seawater is clearly different from that for ultrapure water. Sr^2+^ exchanges alternately with competing ions, so complete breakthrough is difficult to reach, and the maximum dynamic adsorption capacity drops sharply.

In terms of uranium adsorption, the adsorption capacity of amorphous spherical granular titanium phosphate synthesized by Zakutevskyy et al. [84] for UO_2_^2+^ was 4.4 mmol/g (8.8 meq/g). Its adsorption follows an ion-exchange-precipitation mechanism. Since Na^+^ undergoes neither precipitation nor strong complexation, its exchange capacity serves as the baseline for ion exchange. At pH 5.5, the Na^+^ exchange capacity was 2.5 meq/g, whereas the UO_2_^2+^ adsorption capacity reached 8.8 meq/g, far exceeding this value. This confirms that UO_2_^2+^ removal involves both ion exchange and surface precipitation. Under conditions where ion exchange cannot proceed, such as in acidic media where functional groups are nondissociated or in weakly alkaline media where UO_2_^2+^ occurs as carbonate anionic complexes, uranium is removed predominantly by precipitation. This allows treatment of real uranium-containing solutions without optimized conditions.

The ion-exchange capacity of phosphate materials is closely related to their crystal structure. Wu et al. [85] prepared a NASICON-type Na_4_MnAl(PO_4_)_3_ (NMAP) material by combining the sol–gel method with high-temperature sintering. The material has an R-32 space group structure with open ion channels. At pH 3.0, NMAP reached adsorption equilibrium within about 120 min and showed a high adsorption capacity of 670.19 mg/g. In complex water, the distribution coefficient K_d_ exceeded 10^4^ mL/g. DFT calculations indicated that phosphate groups and sodium vacancies are the main binding sites. Detailed characterization further suggested that several interactions are involved in adsorption: ion exchange, surface redox reactions mediated by Mn species, coordination with P=O and M–O groups, and electrostatic attraction. The material remained stable after five adsorption–desorption cycles.

In addition to adsorption performance, stability and selectivity are equally important. Abdel-Galil et al. [86] synthesized cubic crystalline molybdenum-titanium-tungsten phosphate MoTiWPO_4_ (grain size ~51.6 nm, specific surface area 39.5 m^2^/g), which is more stable in acidic and alkaline media. The saturated adsorption capacity for Sr^2+^ was 109.11 mg/g, and the adsorption equilibrium was reached in 60 min. In simulated wastewater containing ^60^Co and ^152^Eu, the removal rate of ^85^Sr reached 90.3%, showing good selectivity.

The synthesis route affects whether phosphate groups remain accessible or become buried in a dense framework, and this accessibility appears more closely related to the binding capacity than the total phosphate content [82,83,84,85]. The aerogel route preserves a mesoporous network, which shortens the mass transfer path and favors high capacities and fast equilibration [82,83]. However, a high capacity alone does not reveal the removal mechanism. When the measured capacity exceeds the ion-exchange capacity of the material, additional mechanisms such as surface precipitation also contribute to removal [84]. In addition, stability under acidic and alkaline conditions and in the presence of radioactive ions is also a relevant criterion when comparing phosphate materials [86].

Across the inorganic material categories reviewed above, the highest reported removal capacities for both Sr^2+^ and UO_2_^2+^ are found for phosphate-based materials, titanosilicates, and titanium-based oxides. Taking the equilibration time as the indicator, some titanates and phosphate aerogels equilibrate fastest, within about 10 min, whereas the other materials generally require tens of minutes to several hours [73,82]. In terms of Sr^2+^ selectivity, titanates and titanosilicates are the most selective types, while phosphate-based materials also show good Sr^2+^ affinity [71,77,82]. For UO_2_^2+^, selectivity relies more on surface complexation and reduction pathways than on size sieving [78,85]. Each type of material also has its own practical advantages: iron oxide-based composites allow magnetic separation [56,57], titanium-based oxides can be coupled with photocatalytic or photoelectrocatalytic reduction [67], titanates and titanosilicates offer high Sr^2+^ selectivity, and phosphate-based materials show strong affinity for both Sr^2+^ and UO_2_^2+^, with some of them also resistant to acid, alkali, or irradiation [83]. In practical use, however, these materials still face several constraints: shaping generally reduces the intrinsic capacity, coexisting cations such as Ca^2+^, Mg^2+^, Na^+^, and K^+^ can lower the selectivity in real waters, and long-term stability, regeneration performance, and column-scale behavior are still insufficiently reported, which makes it difficult to assess their suitability for scale-up. In terms of intrinsic performance, titanates and titanosilicates are the most effective for Sr^2+^, whereas phosphate-based materials and titanium-based oxide composites show greater promise for UO_2_^2+^.

### 4.2. Organic–Inorganic Hybrids

Organic–inorganic hybrid materials are formed by the combination of organic and inorganic components at the molecular scale or nanoscale. In the Class I type hybrids, the organic components are embedded or dispersed in the inorganic network by means of weak interactions such as hydrogen bonds and van der Waals forces. The Class II type hybrids anchor the organic ligand to the inorganic skeleton through covalent bonds, and the structure is more stable, and the functional groups are not easily lost. Sol–gel hybrid materials are mainly prepared by co-hydrolysis-co-condensation or post-grafting [87,88,89].

#### 4.2.1. Phosphonic Acid-Based Hybrids

The phosphonate group (–PO(OH)_2_) has a high charge density. Its two ionizable hydroxyl groups gradually dissociate into –PO_3_H^−^ and –PO_3_^2−^ under different pH conditions. This gives the oxygen atoms a strong negative charge and provides both electrostatic attraction and ion-exchange ability for multivalent cations. The phosphonic acid group also shows chelating coordination recognition for UO_2_^2+^. In contrast, Sr^2+^ adsorption depends mainly on the synergistic effect of electrostatic attraction and ion exchange [90,91,92].

Mu et al. [93] synthesized zirconium phosphonate hybrid materials using ZrOCl_2_·8H_2_O and organic phosphonate ligands. By replacing part of the phosphate groups in the interlayer of α-zirconium phosphate (α-ZrP) with organic phosphonate ligands, the interlayer spacing was significantly enlarged. The maximum Sr^2+^ adsorption capacity given by the Langmuir model was 174.45 mg/g, much higher than that of traditional α-ZrP (55 mg/g). This value was obtained under the conditions of pH 6, 293 K, and an initial concentration range of 50–1000 mg/L (R^2^ = 0.998). When Ce reached about 400 mg/L, the isotherm leveled off, indicating that adsorption was approaching saturation, and the experimental equilibrium capacity (q_e_,_exp_ = 174.25 mg/g) was close to the fitted value.

In addition to layered phosphonates, multi-metal oxide composites also show good adsorption performance. Fares et al. [94] used ZrOCl_2_·8H_2_O and tetraisopropyl titanate (TTIP) as metal precursors and phosphonic acid (PA) as a coupling agent to prepare a TiO_2_–ZrO_2_/PA/AC ternary composite. ZrO_2_ provides a stable high-surface-area skeleton and adsorption sites, TiO_2_ enriches surface hydroxyl active centers, phosphonic acid offers chelation through P–O and P=O groups, and activated carbon (AC) contributes additional pore structure and physical adsorption capacity. The Langmuir model gave maximum adsorption capacities of 467.83 mg/g for Sr^2+^ (310 K) and 171.19 mg/g for UO_2_^2+^ over an experimental concentration range of 50–500 ppm. Kinetically, adsorption follows the pseudo-second-order model, indicating that chemisorption dominates through valence forces between the metal ions and the phosphonate (PA) and metal–hydroxyl (M–OH) groups. The process is endothermic, with capacity increasing at higher temperatures. The Boyd model indicates that intraparticle diffusion is the primary step controlling the rate at which equilibrium is achieved. Dosage experiments further show that Sr^2+^ reaches its removal plateau at a lower dosage, indicating excellent affinity for Sr^2+^.

**Figure 6:**
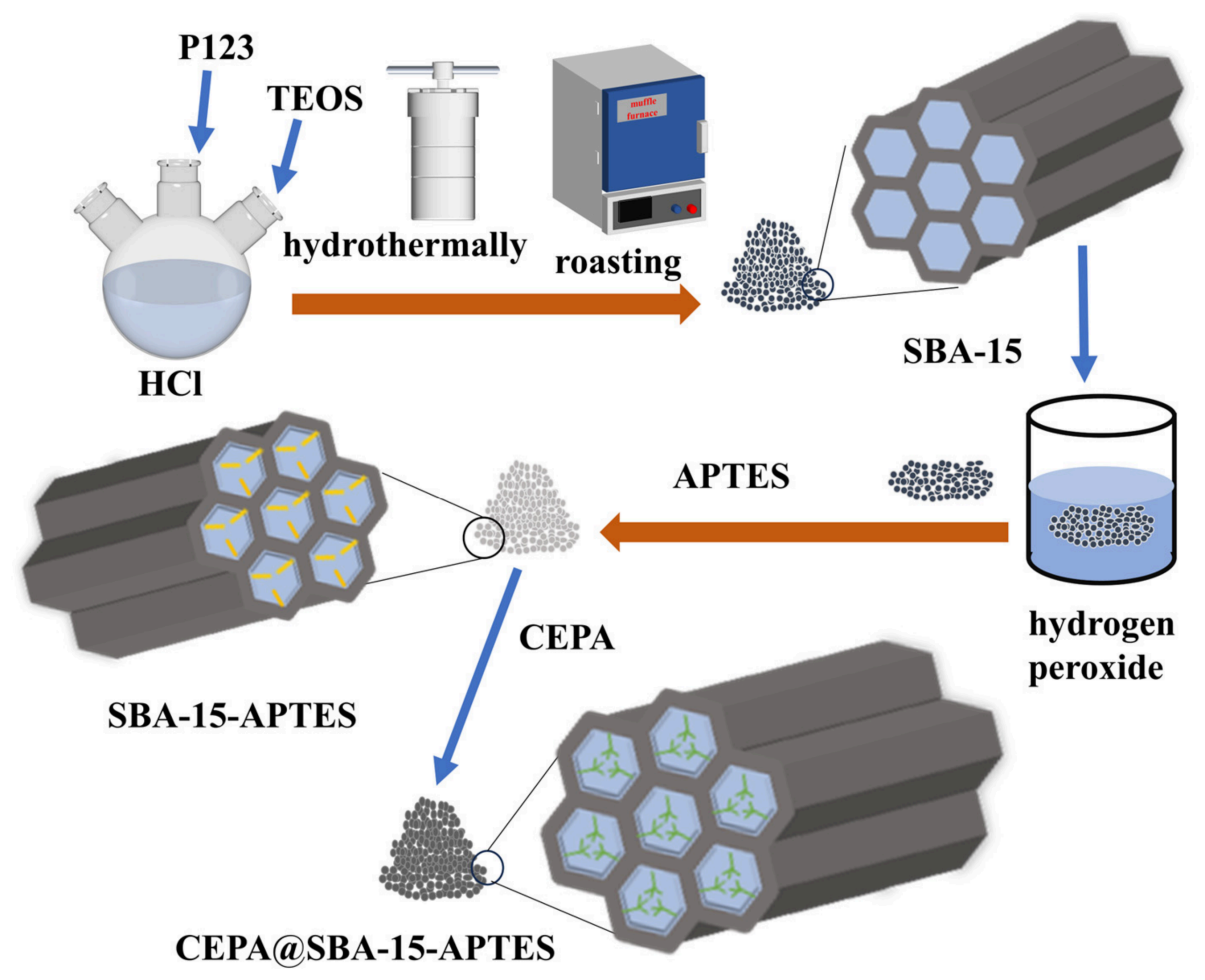
Schematic diagram of CEPA@SBA-15-APTES preparation [95].

In the mesoporous system, Zhang et al. [95] used ordered mesoporous SBA-15 as the substrate, first modified it with APTES (3-aminopropyltrimethoxysilane) for amination, and then grafted 2-carboxyethylphosphonic acid (CEPA) onto the surface by an amidation reaction to prepare the phosphonic acid/amide bifunctional mesoporous silica adsorbent CEPA@SBA-15-APTES (Figure 6). The material has an adsorption capacity for Sr^2+^ over a wide acidity range of 10^−10^–4 M HNO_3_. In 10^−6^ M HNO_3_, equilibrium is reached in 30 min, and the saturation adsorption capacity at 318 K is 112.6 mg/g; in 3 M HNO_3_, equilibrium is reached in 5 min, and the saturation adsorption capacity at 318 K is 71.8 mg/g. Based on experimental and characterization results, the adsorption mechanism involves ion exchange between Sr^2+^ and H^+^ in P–OH in 10^−6^ M HNO_3_ and coordination between Sr^2+^ and oxygen-containing groups (C=O and P=O) in 3 M HNO_3_. In simulated high-level liquid waste containing 15 kinds of ions such as Mo, Pd, U, Y, Cs, and Ru, the separation factor of Sr^2+^ is greater than 90, showing good separation ability.

However, the loading of phosphonate groups is not as high as possible. Dudarko et al. [96] synthesized SBA-15-type silica-tethered phosphonic acid, with diethylphosphatoethyltriethoxysilane (DPTS) chosen for the incorporation of P-containing functional groups. The specific surface area of the optimal adsorption material was 533 m^2^/g, the loading of phosphonic acid groups was 2.0 mmol/g, and the mesoporous pore size was 6.9 nm. The adsorption capacity of UO_2_^2+^ reached 55 mg/g under simulated seawater conditions (pH 8.3). At the same time, we found that when the loading amount of phosphonic acid groups exceeded 2.6 mmol/g, pore blockage and disruption of the mesoscopic structure reduced the adsorption capacity. This indicates that both the density of functional groups and the accessibility of pores need to be considered.

Unlike the above ordered mesoporous powder, Peled et al. [97] prepared a phosphonic acid-functionalized hybrid gel adsorbent (TMOS-NTPH) using TMOS as the silicon source and [nitrilotris(methylene)]tris(phosphonic acid) (NTPH) as the phosphonic acid ligand. ^31^P NMR confirmed that the phosphonate group of NTPH was anchored to the SiO_2_ network through P–O–Si covalent bonds. The adsorption capacity for UO_2_^2+^ was 28.4 μmol/g. The introduction of NTPH accelerated the adsorption of UO_2_^2+^, reaching the maximum capacity within 1 h. In contrast, Cr^3+^ showed a slow ligand exchange rate and a continuous increase in adsorption capacity with time, allowing Cr/U separation by kinetic difference. DFT calculations show that the binding free energy of the phosphonic acid group with UO_2_^2+^ is lower than that with divalent cations such as Ni^2+^, Co^2+^, and Pb^2+^. This theoretically explains the selectivity of the phosphonic acid-functionalized matrix for UO_2_^2+^. Table 2 summarizes the performance comparison of uranium and strontium adsorption materials of phosphonic acid hybrid materials.

Overall, the phosphonic acid hybrids show that a higher phosphonate loading does not necessarily give a higher uptake. Pore blocking and loss of mesoscopic order can reduce the number of accessible sites [96]. The selectivity of phosphonate groups for UO_2_^2+^ over divalent cations has so far been rationalized mainly through DFT calculations. However, direct spectroscopic evidence for the proposed binding mechanism remains limited [97].

#### 4.2.2. Amino/Carboxyl Hybrids

The amino group (–NH_2_) and carboxyl group (–COOH) contain nitrogen and oxygen donor atoms. They coordinate with UO_2_^2+^ and Sr^2+^ through hard–soft acid-base matching [98]. UO_2_^2+^ is a hard acid, while –COOH is a hard base. They can form a stable inner-layer complex. The –COOH group can also remove Sr^2+^ by ion exchange. Upon deprotonation, –NH_2_ forms a stable coordination complex with the borderline acid UO_2_^2+^ through the lone pair electrons of the nitrogen atom. The surface charge can be regulated by the protonation/deprotonation balance, which synergistically enhances the selective recognition of UO_2_^2+^ [99,100].

Chitosan is an ideal organic component for the construction of amino hybrid materials due to its intrinsic ––NH_2_ and–OH groups. Liao et al. [101]. prepared polyethylenimine (PEI)-enhanced phosphoric acid-doped chitosan-silica hybrid aerogel (Pi–P@CSHA) by the sol–gel-freeze-drying technique. The ––NH_2_ and ––OH groups on the molecular chain of chitosan provide complexation sites for UO_2_^2+^. PEI enhances the anti-dissolution stability of the material under acidic conditions by crosslinking, and its abundant NH_2_ groups further increase the density of active sites. Phosphate (PO_4_^3−^) provides a high-affinity specific binding site. The maximum adsorption capacity is 244.9 mg/g at pH 4.0, and the adsorption efficiency is 58.8% at pH 2.0, which is suitable for acidic wastewater treatment.

In chitosanf-based hybrid systems, –NH_2_ is usually the main adsorption site. Liao et al. [102] took a different approach. They synthesized Zn^2+^-doped and––NH_2_-modified TiO_2_–SiO_2_ aerogel (ZN@TS) by the sol–gel method and freeze-drying. The –NH_2_ was introduced by 1,3-diaminopropane. Sr^2+^ capture was driven mainly by ––OH coordination, while –NH_2_ provided secondary chelation, hydrophilicity, and charge regulation. Together with the oxygen vacancies induced by Zn^2+^, these effects enhanced adsorption synergistically. The specific surface area increased from 46.6 to 416.2 m^2^/g. The maximum adsorption capacity for Sr^2+^ reached 87.8 mg/g, 3.3 times that of unmodified TiO_2_–SiO_2_ (26.8 mg/g). Even with 1000 mg/L Na^+^, K^+^, Mg^2+^, and Ca^2+^ coexisting, the exergy efficiency remained above 68.9%.

Li et al. [103] showed another approach: using an amino-containing silane coupling agent as the only precursor to directly introduce amino groups into the SiO_2_ framework. Using bis(trimethoxysilylpropyl)amine (TSPA) as the sole precursor, a Sr^2+^-imprinted hybrid gel was prepared in a one-step sol–gel process. The secondary amine group (–NH–) in the TSPA molecules gets evenly embedded into the SiO_2_ framework during gelation, giving the material the ability to coordinate with Sr^2+^. The adsorption increases with higher pH and is not sensitive to ionic strength or temperature.

Besides silicon-based hybrid systems, this route can also build non-silicon-based hybrid systems. Zhou et al. [104] used ZrCl_4_ and 2,5-dihydroxyterephthalic acid as precursors to synthesize carboxyl-functionalized UiO–66–(COOH)_2_ xerogels. This differs from traditional hydrothermally synthesized crystalline MOF powders. The material keeps the crystalline hybrid network structure of the MOF while achieving a hierarchically porous macroscopic morphology. Its high density of carboxyl groups (–COOH) provides plenty of binding sites. The hierarchical porous structure ensures accessibility to active sites and rapid mass transfer. Its maximum adsorption capacity for UO_2_^2+^ reaches 395 mg/g, higher than ordinary powdered UiO–66–(COOH)_2_ (150 mg/g). It also shows good selectivity for uranium in simulated seawater.

Overall, the uptake is influenced mainly by the surface functional groups, while the inorganic framework mainly provides structural support and site accessibility. Different functional groups play different roles in binding, and depending on the system, they may act synergistically or with one group dominating, so that the same hybrid system can capture UO_2_^2+^ or Sr^2+^ by different routes [101,102]. Different routes of functional-group introduction also change its accessibility [102,103]. In the carboxyl-functionalized system, the high density of –COOH groups provides the binding sites, and their accessibility may be influenced by the hierarchical porous morphology [104].

#### 4.2.3. Other Functional Group-Based Hybrids

In addition to the functional groups mentioned above, functional groups such as crown ethers, oxazolines, and thiol groups can also be introduced into organic–inorganic hybrid frameworks via the sol–gel method, endowing the materials with the ability to selectively recognize specific nuclides.

The nitrogen and oxygen atoms in the amidoxime group (–C(NOH)NH_2_) provide lone pairs of electrons that can form five-membered chelates with the empty orbitals of UO_2_^2+^, thereby enhancing the material’s coordination affinity for uranyl ions. Liao et al. [105] introduced amidoxime-modified hydroxyapatite into a sodium alginate–Ca^2+^ crosslinked system to make composite gel beads. The amidoxime groups provide chelation, while PO_4_^3−^ forms ionic complexes with UO_2_^2+^ through Coulombic attraction. The two groups act synergistically, increasing the adsorption capacity from 308.7 mg/g to 466.0 mg/g and shortening the equilibrium time from 300 min to 120 min. Yang et al. [106] used a sol–gel method combined with a two-step post-grafting approach to dope TiO_2_ with SiO_2_ and graft amidoxime (AO), resulting in amidoxime-functionalized TiO_2_@SiO_2_ composite adsorbents (AO–TiO_2_@SiO_2_). The addition of SiO_2_ effectively suppressed nanoparticle aggregation, resulting in a surface area of 196.6 m^2^/g, while AO grafting introduced multidentate coordination structures on the material surface with high selectivity for UO_2_^2+^. Its maximum adsorption capacity for UO_2_^2+^ reached 648.4 mg/g with an adsorption efficiency of 99.1%, approximately 2.7 times and 1.5 times higher than pure TiO_2_ and TiO_2_@SiO_2_, respectively.

Crown ether-based hybrid materials have unique advantages for selectively separating Sr^2+^, mainly due to the size-matching effect of the crown ether cavity. The cavity diameter of 18-crown-6 (2.6–3.2 Å) closely matches the ionic diameter of Sr^2+^ (about 2.36 Å), allowing selective complexation through ion-dipole interactions [107,108]. Yost et al. [109] physically encapsulated the crown ether ligand 1,4,10,13-tetraoxa-7,16-diazacyclooctadecane-7,16-bis(malonate) (Na_4_oddm) into a SiO_2_ matrix using a simple sol–gel process, and even in the presence of excess Ca^2+^, the Sr^2+^ removal rate still reached 91.4%. Momen et al. [110] prepared SiO_2_-encapsulated 4,4′,(5′)-di-tert-butylcyclohexano-18-crown-6 (DtBuCH18C6) via sol–gel chemistry and found that the Sr^2+^ adsorption amount was not proportional to the crown ether loading, indicating the limitation of physical encapsulation where crown ethers are buried inside the gel and cannot participate in coordination. Bai et al. [111] modified the macrocyclic ligand cis-dicyclohexano-18-crown-6 (cis-DCH18C6) into a monomer containing two silane groups, then co-hydrolyzed and co-condensed it with TEOS, anchoring the crown ether to the SiO_2_ framework through Si–C covalent bonds. The functionalized organosilicas showed favorable adsorption capacity and selectivity for Sr^2+^ in HNO_3_ media; the sample Crown-75 had a Sr^2+^ distribution coefficient of 185.3 cm^3^/g in 1 mol/L HNO_3_. Crown-50 was treated in 1 mol/L HNO_3_, and the carbon loss was less than 3% after more than 5000 min. To test the radiation stability, the Sr^2+^ separation ability of recycled Crown-50 in simulated high-level liquid waste was examined at different absorbed doses. Even at an absorbed dose of 10^5^ Gy over about 1000 min, the carbon loss remained below 4%, and the distribution coefficient showed no obvious decline over the tested dose range. These results indicate that the crown ether groups covalently bonded to the organosilica matrix remained stable in 1 mol/L HNO_3_ and were not noticeably damaged by irradiation, and that the material retained its Sr^2+^ separation ability under these conditions.

The affinity of sulfhydryl (–SH) to UO_2_^2+^ is due to the coordination of sulfur atoms (with U–S orbital hybridization and electron transfer), as well as the electrostatic attraction and coordination synergistic effect of deprotonated thiolate anions (R–S^−^). Although S (soft base) and UO_2_^2+^ (hard acid) are not the optimal pairing in HSAB theory, the covalent nature of the U–S bond contributes significantly to the stability [112,113]. A thiol-functionalized PVA/TEOS/TMPTMS hybrid adsorbent was prepared by Irani et al. [114]. The maximum adsorption capacity of UO_2_^2+^ was 39.08 mg/g at the optimal ratio, and the adsorption capacity did not decrease significantly after 5 cycles.

In addition to the above groups, small organic molecule ligands can also be grafted onto the surface of core-shell structures via the sol–gel method. Sadeghi et al. [115] used Fe_3_O_4_ as the magnetic core and coated it with a SiO_2_ shell through the sol–gel method. Then, the organic molecule quercetin was grafted onto the shell via APS coupling, resulting in the Fe_3_O_4_@SiO_2_-quercetin (QASM) hybrid adsorbent, which can adsorb up to 12.33 mg/g of UO_2_^2+^. Quercetin’s chelation gives the material the ability to selectively extract UO_2_^2+^. Following a similar core-shell design, Li et al. [116] combined the sol–gel method with surface molecular imprinting. Synthesized uranyl-salophen was used as the template and 3-aminopropyltriethoxysilane (APTES) as the functional monomer. A uranyl-imprinted organic polymer layer was coated on Fe_3_O_4_@SiO_2_ to make a core-shell surface-imprinted magnetic chelating adsorbent (UMCA). The size match of the imprinted cavities and the oriented layout of functional groups greatly boost UO_2_^2+^ recognition (relative selectivity coefficient k_0_ = 4.101). The magnetic core also makes magnetic separation very easy.

Taken together, these hybrids rely on different functional groups to bind the target ions. Their selectivity arises from different mechanisms. The type of functional group is not the only variable. How the group is introduced and fixed also affects accessibility and stability. Physically encapsulated ligands can be buried inside the gel and become inaccessible [109,110]. Covalent anchoring keeps the group available and improves stability under irradiation [111]. Selectivity is governed mainly by the match between the ligand cavity or chelate geometry and the target ion [105,111]. It can be further enhanced by imprinting cavities whose size and group orientation match the target [116]. Even when the hard–soft acid-base match is not ideal, a covalent contribution to the metal-ligand bond can still provide effective binding [114].

Across the organic–inorganic hybrids reviewed above, the highest reported UO_2_^2+^ capacities are found for amidoxime-functionalized materials [105,106], while the highest Sr^2+^ capacities are found for phosphonate-based systems [93,94]; amino- and carboxyl-functionalized hybrids give moderate capacities but broader applicability [101,104]. In terms of equilibration time, most hybrids equilibrate within tens of minutes to a few hours. In terms of selectivity, crown-ether-based hybrids are the most Sr^2+^-selective because of cavity-size matching [107,108,109], while amidoxime- and phosphonic acid-functionalized materials show high affinity for UO_2_^2+^ [97,105,106]; amino- and carboxyl-functionalized hybrids are less selective but relatively tolerant of high ionic strength [102,104]. Each type of hybrid also has its own practical advantages: magnetic core-shell hybrids allow magnetic separation [115,116], crown-ether systems tolerate excess Ca^2+^ [109], and phosphonic acid-functionalized mesoporous silicas retain Sr^2+^ selectivity in multi-ion media [95]. Several constraints, however, remain. Coexisting cations in real waters can lower selectivity, and the organic components may be lost during repeated use or disposal. In addition, the long-term stability, regeneration performance, and column-scale behavior of hybrid adsorbents are still insufficiently reported, which makes it difficult to assess their suitability for scale-up. In terms of intrinsic performance, phosphonate-based hybrids are the most effective for Sr^2+^ [93,94], crown-ether-based hybrids are the most selective for Sr^2+^ [109,110,111], and amidoxime-functionalized hybrids are the most promising for UO_2_^2+^ [105,106].

## 5. Mechanisms and Applicability of Adsorbents

### 5.1. Evidence Grading of Reviewed Studies

To evaluate the studies reviewed in Section 4, a four-level evidence scale was developed based on three dimensions: an aqueous matrix, adsorption data, and mechanistic characterization. The aqueous matrix is classified as follows. Complex water refers to real or simulated multi-ion waters, such as tap water, surface water, groundwater, seawater, and simulated high-level liquid waste. A synthetic aqueous solution refers to laboratory-prepared solutions containing the target nuclide, either alone or together with other metal ions; studies that use a synthetic aqueous solution as the base system remain in this category even if they additionally perform coexisting-ion experiments, because such experiments are treated as condition experiments and are reflected in the adsorption-data dimension. The highest level is assigned to studies that use a synthetic aqueous solution as the base system and perform supplementary tests in complex water, because such studies provide both mechanistic information under laboratory conditions and applicability information under complex-water conditions. Because the included studies with supplementary tests in complex water generally provided complete adsorption data and mechanistic characterization, the complex-water studies were not further divided by evidence strength. The criteria for each level are summarized in Table 3 and are based on the common features of most reviewed studies. When a study does not cover one of these dimensions, or discusses it only to a limited extent, its level is assigned by overall judgment rather than by strict correspondence with the table. Each level cumulatively includes the criteria of all lower levels. When a study performs strongly in one dimension but weakly in another, the overall level is assigned on the basis of the combined evidence rather than by the weakest dimension alone.

### 5.2. Structure Regulation

The sol–gel method controls the structure of adsorption materials in two ways. It arranges sites at the molecular scale and controls morphology at the macroscopic scale. These two effects are combined through precursor design and process parameters.

Precursor ratio regulation, organic group modification, and sequential introduction of multiple components can coordinate the polycondensation of precursors with different hydrolysis characteristics. These strategies inhibit phase separation and produce a uniform, stable sol system, providing a time window for the distribution of active sites [55,61]. Its mild conditions are conducive to the retention of functional groups, but the loading amount needs to be regulated by the precursor ratio. Excessive modification will block the pores and reduce the site accessibility [96]. Aging can improve the uniformity of the gel network and promote the uniform dispersion of active components [71]. Subsequent heat treatment can lead to the transformation of specific crystal structures such as layered and perovskite types, accompanied by morphological evolution [55,71,79]. The specific surface area alone, however, does not determine the adsorption performance. The adsorption performance is governed by the density and accessibility of surface active sites [63]. Therefore, a material with a lower specific surface area can still perform better. This can occur when it has a higher surface hydroxyl density and a larger mesopore size [63]. The synthesis parameters therefore largely govern which active sites are formed and how densely they are distributed. This provides the basis for the subsequent adsorption mechanism.

Pore structure directly affects mass transfer. Micropores offer enrichment sites but cause high diffusion resistance [77]. Pores that are too small increase ion diffusion resistance. A three-dimensional interconnected mesoporous network, however, can greatly shorten the mass transfer path, enabling adsorption equilibrium within minutes [74,82]. The large hole has a limited contribution to the capacity, mainly to reduce the pressure drop and support the molding [77]. Although the interlayer channels of layered materials have selective screening ability for ions, too narrow spacing will limit the diffusion rate [82,93], which indicates that the pore design needs to be considered comprehensively between specific surface area utilization and mass transfer efficiency. In addition to the active-site density described above, these structural features determine the accessibility of the active sites to the target ions. They also influence which adsorption mechanism predominates, such as surface complexation, ion exchange, or precipitation.

Diversified macroscopic morphologies can be obtained by the sol–gel method through different process paths. Aerogel monolithic materials with the potential for adsorption-separation integration can be obtained by adjusting the drying method [101,102]; combined with a core-shell structure, a composite adsorbent suitable for column operation can be obtained [73]. Combining it with the gel-forming method directly produces millimeter-scale microspheres with a spherical shape favorable for column packing [66]. These examples show that the sol–gel method has good process compatibility and allows the macroscopic morphology of the adsorbent to be controlled in different ways, thereby offering potential for engineering applications such as fixed-bed and membrane systems.

Taken together, the synthesis parameters, active sites, structure, and morphology discussed above jointly determine the adsorption mechanism and, through it, the selectivity and engineering applicability of the material. Synthesis conditions control which active sites are formed; the pore and interlayer structure determine whether these sites can be reached by the target ions; and the resulting binding mode influences both selectivity and engineering performance. Overall, performance is governed by the combined effects of these variables rather than by any single parameter.

### 5.3. Adsorption Mechanisms

**Figure 7:**
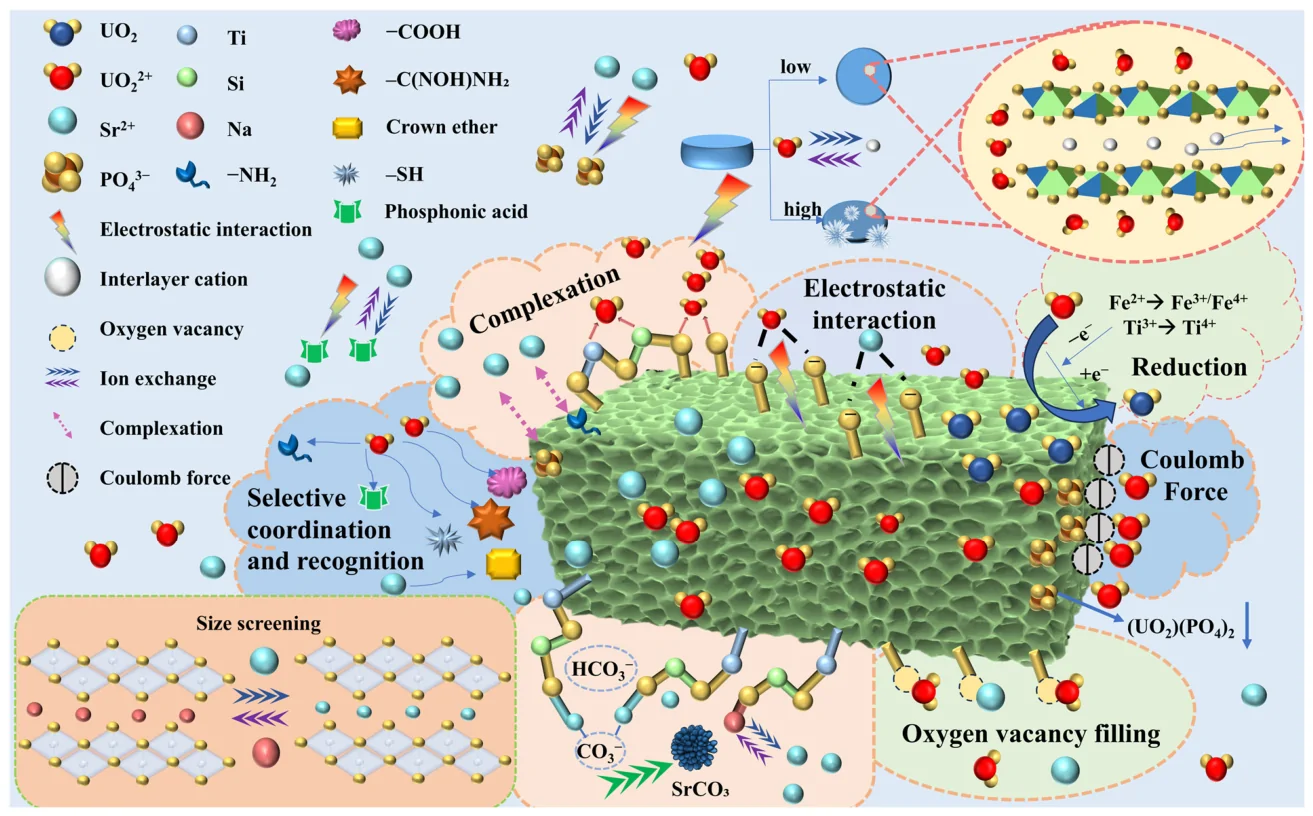
The main adsorption mechanism of UO_2_^2+^ and Sr^2+^ on the materials prepared by the sol–gel method.

The removal and sequestration of Sr^2+^ and UO_2_^2+^ by sol–gel materials involve several interfacial processes (Figure 7).

Adsorption and complexation: The surface ≡M–OH is deprotonated to ≡M–O^−^ at a suitable pH. The cation is then captured by electrostatic interaction in the outer sphere. This interaction is restricted by the isoelectric point and pH. The Coulomb attraction is the strongest when the surface is fully deprotonated. Under strongly acidic conditions, protonation leads to repulsion [56,78,105]. The surface hydroxyl oxygen can also coordinate directly to the metal center. This forms an inner-sphere complex (≡M–O–UO_2_^2+^). The process involves coordination of oxygen lone pair electrons to the metal’s empty orbital. It is accompanied by proton release. The binding strength is higher than that of electrostatic adsorption. It is also insensitive to ionic strength. The efficiency depends on the accessible hydroxyl density [56,62]. In addition, surface PO_4_^3−^ binds Sr^2+^ mainly through ionic interactions [82]. Functional groups such as amidoxime and crown ether provide selective binding through chemical recognition [105,111]. Molecular imprinting enables directional capture through topological holes [116]. These strategies can be combined with geometric screening and cavity size matching of the pores or interlayers. This gives the material the ability to recognize and selectively fix specific nuclides.

Ion exchange: The exchangeable cations in the framework can be replaced by Sr^2+^ and UO_2_^2+^. Titanate shows a size-screening effect on Sr^2+^. The selectivity depends on the matching of hydrated ion radius, charge density, and channel or interlayer spacing [71,72,78]. The introduction of interlayer species can expand the interlayer spacing and relax the skeleton. This reduces the exchange barrier [93].

Precipitation and coprecipitation: UO_2_^2+^ is enriched in the interlayer or channel. It then migrates to the surface for crystallization and precipitation. SrCO_3_ deposition can also form when Sr^2+^ is partially supersaturated [77]. This changes reversible exchange into irreversible fixation. The PO_4_^3−^ on the framework or surface can react with UO_2_^2+^ to form insoluble uranyl phosphate [84].

Reduction and immobilization: Interfacial electron transfer reduces UO_2_^2+^ and forms insoluble UO_2_. Electrons can be provided by lattice electron transfer of variable-valence metal centers, photogenerated electrons or superoxide radicals, or an applied electric field. These electrons form UO_2_ or hydrated oxides deposited on the surface [55,67]. Lattice oxygen vacancies can directly capture Sr^2+^ or UO_2_^2+^. The cation enters the defect site, stabilizes the lattice, and achieves bulk-phase fixation [64,102].

### 5.4. Competing-Ion Effects and Selectivity

The following discussion focuses on the competing ions most frequently examined in the studies reviewed here—Na^+^, K^+^, Ca^2+^, Mg^2+^, CO_3_^2−^ and SO_4_^2−^. Real radioactive wastewater contains many other ionic species, but their effects are rarely quantified in the literature and are therefore not discussed in detail here.

When Na^+^ and K^+^ coexist with Sr^2+^, their smaller hydrated radii (Na^+^, 2.76–3.58 Å; K^+^, 2.32–3.30 Å) relative to that of Sr^2+^ (4.10–4.20 Å) weaken their ability to compete for the same binding sites, and their low positive charge also weakens their electrostatic interaction with negatively charged surface groups, so their interference with Sr^2+^ adsorption is weaker than that of Ca^2+^ and Mg^2+^ [102]. Ca^2+^ and Mg^2+^ compete more strongly, carrying the same charge as Sr^2+^ and having similar ionic radii; Ca^2+^ is the stronger competitor, while Mg^2+^ enters narrow interlayer or channel spaces less readily owing to its larger hydrated radius [72]. For UO_2_^2+^, Ca^2+^ and Mg^2+^ interfere less at low pH but more at higher pH [67], whereas Na^+^ and K^+^ generally have a weaker effect, and several materials retain high UO_2_^2+^ uptake in the presence of large amounts of Na^+^ and K^+^ [56,67]. Anions have a more pronounced effect on UO_2_^2+^ than on Sr^2+^, with CO_3_^2−^ showing the strongest influence, SO_4_^2−^ varying with concentration, and Cl^−^ and Br^−^ having only a minor effect [64,67]. For Sr^2+^, anions generally have a limited influence, although carbonate species can promote the formation of SrCO_3_ heterophase on the surface [77].

Based on the studies reviewed here, the responses to competing ions differ among material categories. Titanates and titanosilicates both undergo Na^+^ exchange, but their responses to other competing cations differ. For titanates, Ca^2+^ and Mg^2+^ interfere relatively strongly, whereas K^+^ is relatively well tolerated. For titanosilicates, UO_2_^2+^ selectivity is still retained in the presence of various interfering ions [78]. Phosphate-based materials are less affected by Na^+^, K^+^, and Mg^2+^ than by Ca^2+^, and several phosphate frameworks remain effective in seawater and other complex waters [76,77]. Titanium-based oxides are relatively insensitive to common competing cations but relatively strongly affected by CO_3_^2−^ and SO_4_^2−^ [67]. Iron oxide-based composites rely on the shell chemistry rather than the iron oxide core, and their preference can shift from UO_2_^2+^ to Sr^2+^ depending on the shell composition [58,59]. Among organic–inorganic hybrids, crown-ether-based systems tolerate excess Ca^2+^ better than most inorganic adsorbents through cavity size matching with Sr^2+^ [109], phosphonic acid-functionalized mesoporous silicas retain Sr^2+^ selectivity in multi-ion media [95], and amino-functionalized hybrids maintain an efficiency above 68.9% under high Na^+^, K^+^, Mg^2+^, and Ca^2+^ concentrations [96]. Overall, materials combining a size-selective binding environment with a stable anchoring mode, such as layered titanates and crown-ether-functionalized hybrids, show the best selectivity under realistic competing-ion conditions [71,109].

In simulated seawater or studies on real waters, Ca^2+^ and Mg^2+^ are often far more concentrated than Sr^2+^ and occupy many of the exchange sites; at very high concentrations, Na^+^ may also compete for exchange sites and reduce adsorption performance, whereas K^+^ generally has a smaller effect [73,102]. For UO_2_^2+^, CO_3_^2−^ and SO_4_^2−^ can form large uranyl complexes that are less readily complexed or reduced at the surface [67]. These effects are more pronounced in seawater and high-level liquid waste [73,95].

### 5.5. Regeneration and Disposal

For the materials reviewed here, regeneration and spent-adsorbent management have rarely been reported in a systematic way. For inorganic materials, acidic eluents such as HNO_3_ are frequently used [64,78,114]. For organic–inorganic hybrids, chelating agents such as ethylenediaminetetraacetic acid are more common [57]. In addition, Na_2_CO_3_ and H_2_O_2_ are also commonly used for inorganic materials [64]. The desorption efficiency of these eluents varies with the target ion and the material. Chelating eluents are efficient. However, they mobilize target and competing ions. They also generate metal-ligand-rich regenerants [117]. The regenerants generated from chelating eluents require recovery, destructive treatment, or controlled recycling before discharge.

Reported cycle numbers are mostly limited to four to six. Capacities typically remain at 85–90% of the initial value [55,64,67]. For organic–inorganic hybrids, however, only a few studies report cycling performance. The loss of organic functional groups during repeated use also remains largely unquantified. In addition, eluent type, cycle number, and capacity loss are reported in different ways across studies. Therefore, regeneration performance cannot yet be compared on a common basis.

Current regeneration studies rarely quantify the radionuclide concentration in the desorption solution. As a result, the composition and activity of the secondary waste remain largely unknown. The final management of spent adsorbents is also discussed in only a few cases, for example, through sintering into ceramic forms [71]. The long-term stability and leach resistance of such waste forms have rarely been evaluated. This is a particular concern for organic–inorganic hybrids, because their organic components may decompose or leach during disposal.

Current studies pay much less attention to radiation stability than to chemical stability. For adsorbents intended for radioactive wastewater treatment, resistance to ionizing radiation is also important. This is because radiolysis can alter the inorganic framework and the organic components. However, only a few studies have examined radiation stability or tested materials under γ irradiation [111]. For most materials, the effect of γ irradiation on framework integrity and adsorption performance remains largely unknown. Radiation stability is particularly important for organic–inorganic hybrids. Their organic components are more susceptible to radiolytic degradation, yet such studies remain scarce.

## 6. Conclusions and Outlook

This paper reviews the research progress of the sol–gel method in preparing uranium and strontium adsorption materials. This method offers a flexible platform for designing high-performance adsorbents, thanks to its uniform composition, mild conditions, and controllable morphology. Inorganic materials capture uranium and strontium through mechanisms with different emphases. However, they share one thing in common: selective fixation through the chemical affinity between specific active sites and target ions. Organic–inorganic hybrid materials achieve the targeted capture of uranium and strontium through functional group anchoring and synergistic coordination.

From the perspective of practical application, titanate- and titanosilicate-based materials are the most promising for Sr^2+^ removal, because their high selectivity and reversible ion exchange allow efficient Sr^2+^ uptake even in the presence of competing cations, and their inorganic framework avoids the loss of organic components during long-term use. For UO_2_^2+^ removal, phosphonate- and amidoxime-functionalized hybrids are the most promising, because their high capacity and tunable coordination environments allow them to be adapted to different wastewater compositions, but their long-term stability and regeneration behavior still need to be demonstrated at scale.

However, several barriers still prevent their application to real radioactive wastewater. The main technical barriers are as follows: shaping generally reduces the intrinsic capacity, and the mechanical strength of many shaped adsorbents is insufficient for long-term column operation; coexisting ions also interfere, since Ca^2+^ and Mg^2+^, which are far more concentrated than Sr^2+^ in seawater and real waste, compete for the same binding sites and reduce Sr^2+^ selectivity, while CO_3_^2−^ and SO_4_^2−^ form soluble uranyl complexes that keep UO_2_^2+^ in solution and hinder its complexation or reduction at the surface; long-term stability under γ irradiation and repeated adsorption–desorption cycles is rarely evaluated beyond a few cycles, and the fate of radionuclides and reagents during regeneration is usually not quantified; most reported capacities and selectivities are obtained in single-ion or simulated solutions rather than real radioactive wastewater; and preparation cost and batch-to-batch reproducibility have not been systematically assessed.

Future work should proceed in two stages. Before pilot-scale implementation, several experimental and verification steps are required: optimizing the shaping route and the in-situ site density and pore structure to balance capacity, mass transfer, and mechanical strength; reducing preparation cost through cheap inorganic precursors and drying methods suitable for microspheres, fibers, or monolithic blocks; improving selectivity against coexisting ions through multi-site coordination, ion imprinting, or dynamic ligand design; establishing complete mass balances for radionuclides and reagents during regeneration; evaluating capacity retention, structural stability, and radiation stability over multiple cycles; and assessing batch-to-batch reproducibility. Beyond these steps, further research should focus on regeneration, long-term reusability, scale-up, process integration, and spent-adsorbent disposal. Milder and more selective eluents should be developed for regeneration, and cyclability should be tested over many more cycles than currently reported. For scale-up, the synthesis should be simplified and made compatible with continuous production. For process integration, the compatibility between the adsorbent and the coupled process remains to be clarified, and sol–gel-derived adsorbents could be integrated with membrane separation and photocatalytic/electrochemical processes to enable continuous operation or to combine adsorption with reductive immobilization of UO_2_^2+^. For spent-adsorbent disposal, the options depend on the material and the loaded radionuclide: some adsorbents can be regenerated and reused, others may allow recovery of valuable components, and those that cannot be reused or recovered should be converted into stable ceramic, glass-like, or other solid forms to ensure the long-term immobilization of the captured radionuclides.

## Figures and Tables

**Table 1:** Adsorption properties of titanium oxide materials for UO_2_^2+^ and Sr^2+^ ions.

Material	Radionuclides	q_e_ (mg/g)	pH	Equilibration Time (min)	Surface Area (m^2^/g)	Pore Size (nm)	Selectivity	Adsorption Efficiency	Ref.
**TiO_2_**	UO_2_^2+^	NR	9	240	NR	NR	NR	NR	[61]
TiZr22-700	UO_2_^2+^	40.2	3.8	NR	52 ± 1	13.8 ± 0.1	NR	NR	[63]
**GO@Ti_x_Al_1−x_O_y_**	UO_2_^2+^	614.6	4	60	NR	NR	vs. Cd^2+^, Cu^2+^, Ba^2+^, Co^2+^	5 cycles, >90%	[64]
4C–TiO_2_	Sr^2+^	204.4	7	50	379.9	2.4–4.4	NR	5 cycles, ND	[65]
**ZrO_2_–TiO_2_**	Sr^2+^	28.01	ND	ND	489.7	2.48	NR	NR	[66]
TiO_2_/WO_3_@NF	UO_2_^2+^	NA	3	180	64.08	3–5	vs. Na^+^, K^+^, Mg^2+^, Ca^2+^ Cl^−^, Br^−^, SO_4_^2−^, CO_3_^2−^	6 cycles, 87%	[67]

Note: NR refers to data that were not reported; ND refers to data that were mentioned but not specified; NA refers to data that are not applicable to the material or system.

**Table 2:** Adsorption properties of phosphonic acid-based hybrid materials for UO_2_^2+^ and Sr^2+^.

Material	Radionuclides	q_e_ (mg/g)	pH	Equilibration Time (min)	Surface Area (m^2^/g)	Pore Size (nm)	Selectivity	Adsorption Efficiency	Ref.
**ZrP–ATMP**	Sr^2+^	174.45	6	90	NR	NR	NR	NR	[93]
TiO_2_–ZrO_2_/PA/AC	Sr^2+^, UO_2_^2+^	Sr^2+^: 467.83 UO_2_^2+^: 171.19	NR	120	250.3	NR	NR	NR	[94]
**CEPA@SBA-15-APTES**	Sr^2+^	112.6	NR	30	75.1	4–50	vs. Na^+^, K^+^, Ca^2+^, Mg^2+^	NR	[95]
DPTS-10:2	UO_2_^2+^	54.5	8.3	NR	533	6.9	NR	NR	[96]

Note: NR refers to data that were not reported; ND refers to data that were mentioned but not specified; NA refers to data that are not applicable to the material or system.

**Table 3:** Four-level evidence scale.

Level	Aqueous Matrix	Adsorption Data	Mechanistic Characterization	Refs.
**Level I**	Synthetic aqueous solution with complex-water tests	Isotherms, kinetics, and condition experiments are all available, and both isotherms and kinetics are fitted with models.	Mechanistic evidence that directly proves the adsorption mechanism with clear conclusions on coordination, bonding, or valence change, or that strongly supports it with limited inference.	[56,73,82],
				[83,85,86],
				[95,101,102,104,111,116]
Level II	Synthetic aqueous solution	Isotherms, kinetics, and condition experiments are all available, and both isotherms and kinetics are fitted with models.	Mechanistic evidence that directly proves the adsorption mechanism with clear conclusions on coordination, bonding, or valence change, or that strongly supports it with limited inference.	[55,64,67],
				[71,72,74],
				[77,78,105],
				[106]
**Level III**	Synthetic aqueous solution	Isotherms, kinetics, and condition experiments are all available, or partially available.	Routine characterization of phase, morphology, surface area, or elemental composition only, which cannot directly prove the adsorption mechanism.	[54,57,61],
				[63,65,66],
				[79,93,94],
				[96,97,114],
				[115]
Level IV	Synthetic aqueous solution	Isotherms, kinetics, and condition experiments are all available, or partially available.	Characterization absent or minimal	[84,103],
				[109,110]

## Data Availability

No new data were created or analyzed in this study.

## Supplementary Materials

The following supporting information can be downloaded at https://www.mdpi.com/article/10.3390/gels12100891/s1, Supplementary Text S1.
