# Supplementary material for: Recent Advances in Novel Adsorbents Prepared by the Sol–Gel Method for the Removal of Uranium and Strontium from Radioactive Wastewater: A Review
Source: Gels. 2026 Oct 1;12(10):891. doi: 10.3390/gels12100891 (PMC13704472; doi:10.3390/gels12100891)
Supplement: Supplementary file 1 [file gels-12-00891-s001.zip › gels-4533274-supplementary.pdf]

Supplementary Materials

for

Recent Advances in Novel Adsorbents Prepared by Sol–Gel Method for the Removal of Uranium and Strontium from Radioactive Wastewater: A Review

Jiayi Jiang [1], Jiaxue Zhao [1], Miaoling Chen [2], Diyun Chen [1,*] and Minhua Su [1,*]

[1] School of Environmental Science and Engineering, Guangzhou University, Guangzhou 510006, China

[2] Center for Water Research, Advanced Institute of Natural Sciences, Beijing Normal University, Zhuhai, 519087, China

* Corresponding author: cdy@gzhu.edu.cn; mhsu@gzhu.edu.cn

Supplementary Text S1

The literature search was performed in August 2026 using the Web of Science Core Collection, supplemented by Google Scholar and the CNKI database, covering publications from 2000 to August 2026. The search string used in the Web of Science Core Collection was TS = (("sol-gel" OR "sol gel") AND ("uranium" OR "strontium") AND (material keywords)), where TS denotes the topic field.

The same terms were used as keywords in Google Scholar, without field tags: (“sol-gel, sol gel, uranium, strontium, material keywords”).

For CNKI, the search was performed through the advanced search interface. Because the CNKI advanced search limits the number of terms and logical combinations allowed in a single row, the search terms were entered separately with their respective fields. The full-text field was used for the sol-gel terms ("sol gel" OR "sol-gel") because the preparation method often appears in the main text and the subject field retrieved fewer relevant records. The subject field was used for the target nuclide ("uranium" or "strontium") and for the material terms. The search logic was: (full text = "sol gel" OR "sol-gel") AND (subject = "uranium") AND (subject = "iron oxide"); the same query was repeated by replacing "uranium" with "strontium", and by replacing "iron oxide" one at a time with the other material terms.

The material keywords were grouped by material category as follows:

- Iron oxides: "iron oxide" OR "magnetite" OR "maghemite" OR "Fe_3O_4" OR "Fe_2O_3"
- Titanium oxides: "titanium oxide" OR "TiO_2" OR "titania"
- Titanates: "titanate" OR "sodium titanate"
- Titanosilicates: "titanosilicate" OR "silicotitanate"
- Metal phosphates: "metal phosphate" OR "phosphate"
- Phosphonic acid: "phosphonic acid" OR "phosphonate"
- Amino: "amino" OR "amine"
- Carboxyl: "carboxyl" OR "carboxylic"

- Amidoxime: "amidoxime"
- Crown ether: "crown ether"

Studies were screened using the following criteria. Studies were included if they (1) prepared uranium or strontium adsorbents by the sol-gel method or sol-gel-derived routes, (2) reported adsorption, ion exchange, or related removal performance for UO_2^{2+}, Sr^{2+}, or both, and (3) matched the material categories discussed in this review, i.e., inorganic materials and organic-inorganic hybrids. Studies that did not report key experimental conditions or adsorption performance data, and studies that reported the same material system with only minor variations, were excluded. Titles and abstracts were first screened for relevance, and the full texts of potentially relevant records were then assessed. After removing duplicates and applying the criteria above, 39 representative studies were retained for detailed discussion.
